# Engineered Activated Carbons for Multi-Radionuclide Removal from Real Low-Level Radioactive Wastewater

**DOI:** 10.3390/nano16161040

**Published:** 2026-08-21

**Authors:** Mumtaz Khan, Muqaddus Usman Bhurgri, Mazhar Iqbal Zafar, Jie Niu, Aiza Zafar, Penghua Hu

**Affiliations:** 1 Beijing Research Institute of Chemical Engineering and Metallurgy, China National Nuclear Corporation, Beijing 101149, China; 2 Department of Environmental Science, Faculty of Biological Sciences, Quaid-i-Azam University, Islamabad 45320, Pakistanmzafar@qau.edu.pk (M.I.Z.);

**Keywords:** radionuclides, activated carbon, adsorption isotherms, adsorption kinetics, adsorption capacities

## Abstract

Low-level radioactive wastewater requires effective treatment to minimize risks to human health and the environment. This study evaluated the performance of activated carbons derived from regional biomass (Gurgurey, Pine, and Debregeasia woods) and Thar coal for the simultaneous removal of radionuclides (^137^Cs, ^134^Cs, ^124^Sb, and ^122^Sb) from real low-level radioactive wastewater. The activated carbons were characterized by proximate analysis and Fourier Transform Infrared Spectroscopy (FTIR), and their adsorption performance was investigated through batch experiments followed by kinetic and isotherm modeling. Among the investigated materials, activated carbons derived from Gurgurey wood, Pine wood, and Thar coal exhibited superior physicochemical properties, including low moisture (3.8–4.8%), low volatile matter (10.7–15.3%), high fixed carbon content (77.8–84.3%), and adsorption capacities ranging from 205.6 to 251.9 Bq g^−1^. In contrast, Debregeasia-derived activated carbon showed the poorest performance owing to its high moisture, volatile matter, and ash contents, resulting in adsorption capacities of only 53.4–161.2 Bq g^−1^. FTIR analysis confirmed the presence of phosphate-containing functional groups on all biomass-derived activated carbons except Thar coal. The adsorption data were best described by the pseudo-second-order kinetic model and the Langmuir isotherm, indicating that chemisorption and monolayer adsorption were the dominant removal mechanisms. These findings demonstrate that activated carbons derived from Gurgurey wood, Pine wood, and Thar coal are promising low-cost adsorbents for the simultaneous removal of multiple radionuclides from real low-level radioactive wastewater and provide a sustainable approach for radioactive wastewater treatment.

## 1. Introduction

Nuclear power has contributed significantly to the production of clean energy, green electricity production, and “Net Zero Emissions” (NZE) [1]. As global energy dynamics are transitioning towards clean and sustainable energy systems, so too is nuclear power becoming a focal point of this strive [2]. Although nuclear power has many advantages, this clean and cheap power is a source of 10,000–11,000 metric tons of spent nuclear fuel per annum globally [3]. Fuel generation and nuclear fission generate a wide range of radionuclides: ^129/131^I, ^137^Cs, ^79^Se, ^127^Xe, ^90^Sr, ^99^Tc, ^235^U, ^85^Kr, ^122^Sb, etc. This gives rise to many public health, geological, and environmental concerns, creating a wide range of issues [4]. The present study focuses on adsorption treatment of radioactive materials such as ^137^Cs, ^134^Cs, ^124^Sb, and ^122^Sb in wastewater.

Isotopes of cesium (^137^Cs, ^134^Cs) are fission products that pose significant difficulties from a public health and environmental safety perspective [5]. Cesium has a rapid assimilation ability in biological systems due to its high solubility and chemical analogy to alkali metals (e.g., Na^+^ and K^+^). In the body, it can be readily distributed and accumulated in different organs, becoming a long-term radioactive risk [6]. Moreover, ^137^Cs remains in the environmental matrix for centuries, as its degradation can be accomplished by critical, complex, and highly efficient separation methods [7]. The sequestration of ^137^Cs is difficult in radioactive wastewater due to the presence of various mimicking cations that compete for selectivity. Therefore, efficient cesium removal requires highly selective adsorbents possessing appropriate surface functional groups, particularly phosphate (PO_4_^3−^), hydroxyl (–OH), and carboxyl (–COOH) groups, which provide strong and selective binding sites. In recent years, engineered, porous carbon-based adsorbents have emerged as promising materials for the treatment of radioactive wastewater because of their high surface area, tunable surface chemistry, and excellent adsorption performance [8].

On the contrary, antimony is a metalloid that exists at different oxidation states (i.e., 3 or 5), making its sequestration more complex and critical to achieve [9]. The varying oxidation states of antimony are a result of neutron activation in reactor coolant [10]. This results in inactive antimony isotopes (^121^Sb and ^123^Sb), which are anionic in nature and difficult to remove from traditional cationic exchange resins [11]. The antimonite Sb(3) form is more toxic and resistant than the antimonate Sb(V) form. Sb is listed as a cancer-causing contaminant and causes cardiometabolic issues such as diabetes, heart failure, obesity, and hypertension [12]. In aqueous media, Sb species form oxyanions that have high mobility towards groundwater and are resistant to cationic removal matrices [13]. Therefore, there is a dire need for such an adsorbent that has the ability to accomplish critical coordination and complexation in wastewater.

In real nuclear wastewater matrices, the challenge of radioactive decontamination is rarely limited to isolated target radionuclides. Instead, targeted isotopes such as (Cs 137, Cs 134) and (Sb 124, Sb 122) coexist within highly complex backgrounds comprised of competing background electrolytes (e.g., high concentrations of ${\mathrm{Na}}^{+}$, $K^{+}$, ${\mathrm{Ca}}^{2+}$, and ${\mathrm{Mg}}^{2+}$), non-radioactive heavy metals, and persistent dissolved organic matter (DOM). These background matrix components introduce severe thermodynamic and kinetic competition for active surface sites on traditional adsorbents. High-salinity co-existing ions can screen electrostatic surface charges or outcompete alkali metal radionuclides like ${\mathrm{Cs}}^{+}$ for ion-exchange sites due to sheer concentration advantages. Concurrently, co-existing heavy metals and organic foulants can coordinate with surface functional groups, causing active site masking or pore blockage. Achieving highly selective multi-radionuclide sequestration under these competitive scenarios requires advanced interfacial engineering designed to distinguish specific ionic potentials and hydration radii, a feat that remains a critical hurdle for developing cost-effective, real-matrix remediation frameworks [14,15].

There are multiple technologies by which low-level radioactive waste is being treated conventionally. These technologies involve chemical precipitation, membrane filtration, ion exchange, and evaporation [16]. Though these methods have good decontamination factors, they are not yet sustainable and have technological shortcomings [17]. Chemical precipitation is an effective process for treating radioactive waste, but it produces a large quantity of by-product (radioactive sludge) that subsequently requires more steps for stabilization and disposal [18]. Evaporation is an energy-intensive process that requires a high amount of power for volume reduction and produces a volatile form of radioactive waste [19]. Ion-exchange resins are an effective technology, as they can offer selectivity and are energy-efficient [20]. However, saturation of active sites and post-treatment of the resin are among the biggest challenges. Membrane adsorption has evolved with respect to its functionalization and selectivity, yet the issue of membrane fouling remains unsolved [21]. In particular, when a concentrated amount of radioactive brine has to be treated, the practicality of the membrane becomes zero [22]. Due to these operational impediments, there is a research and practical shift towards adsorption [23]. It generally refers to utilizing porous substances in an aqueous medium to remove radioactive waste. The mechanism usually entails electrostatic interactions, size selectivity, and ion exchange [24].

In recent years, a wide variety of sustainable alternative matrices have been explored; for instance, the utilization of microbial systems like *Aspergillus flavus* biomass has shown notable proficiency in the biosorption of dangerous fission products like Cs and Sr from aqueous streams [25]. Similarly, raw agricultural waste biopolymers, such as *Opuntia ficus indica*, have been successfully benchmarked for the highly efficient comparative removal of stubborn dye molecules like Methylene Blue from polluted waters, underscoring the potential of chemically rich natural architectures [26].

Beyond cesium and antimony radiopotentials, the removal of radiostrontium isotopes, such as Sr 85 or Sr 90, represents another critical challenge in low-level radioactive waste management. Although pristine or physically activated carbons often exhibit limited affinity for the highly hydrated divalent strontium ion (${\mathrm{Sr}}^{2+}$), chemical modifications offer a viable pathway to enhance its sequestration. The literature demonstrates that introducing specific oxygen-containing surface functionalities (such as carboxylic, phenolic, and lactone groups) through acid oxidation or grafting specific chelating agents onto the carbon matrix can significantly boost ${\mathrm{Sr}}^{2+}$ immobilization via enhanced ion-exchange and surface complexation mechanisms. Although the direct evaluation of Sr 85 falls outside the experimental scope of the current study, the successful surface functionalization observed herein—such as the inherent integration of phosphate groups on the biomass-derived activated carbons—suggests that these regional precursors hold strong chemical potential for subsequent modification targeted at multi-valent alkaline earth radionuclei like Sr 85.

This research focuses on developing carbon-based (biomass) adsorbents that have high decontamination coefficients and strong adsorption capacities and affinities [27]. Activated carbon (AC) is a widely used adsorbent that has high porosity, a large surface area, (500–1500 m^2^/g), stability, inertness, chemical viability, and critical pore specification [28]. To enhance the porosity and efficiency of AC further, activation agents (such as ZnCl_2,_ H_2_SO_4_, H_3_PO_4_, HNO_3_, NaOH, or KOH) are added during synthesis [29]. The main agenda of adding an activating agent is to increase the porosity and volume of the adsorbent matrix [30]. For this research, phosphoric acid (H_3_PO_4_) is used as an activating agent. It offers improved grids in the structure and better decomposition when carbonized [31].

This research is novel and innovative because it employs an adsorption approach on real radioactive wastewater, while prior literature has employed adsorptive removal of radioactive isotopes in a simulated laboratory setting using stable isotopes. This research study has successfully engineered carbon-based adsorbents with a hierarchical pore framework with oxygen- and phosphate-rich functional groups. This work has not merely synthesized the adsorbent but offered its functionality for the treatment of multi-isotope decontamination.

This research work focuses on the remediation of Cs and Sb radioisotopes using activated carbons in radioactive wastewater. The adsorbents are synthesized through chemical modification of agricultural biomass and coal applied to radioactive wastewater collected from Pakistan Research Reactor (PARR-1), Islamabad. After chemical optimization of activated carbon, the structural and adsorption properties are studied by FTIR. The work explores the relationship between fixed carbon content, phosphate surface groups, and adsorption capacity.

## 2. Materials and Methods

### 2.1. Materials

**Figure 1:**
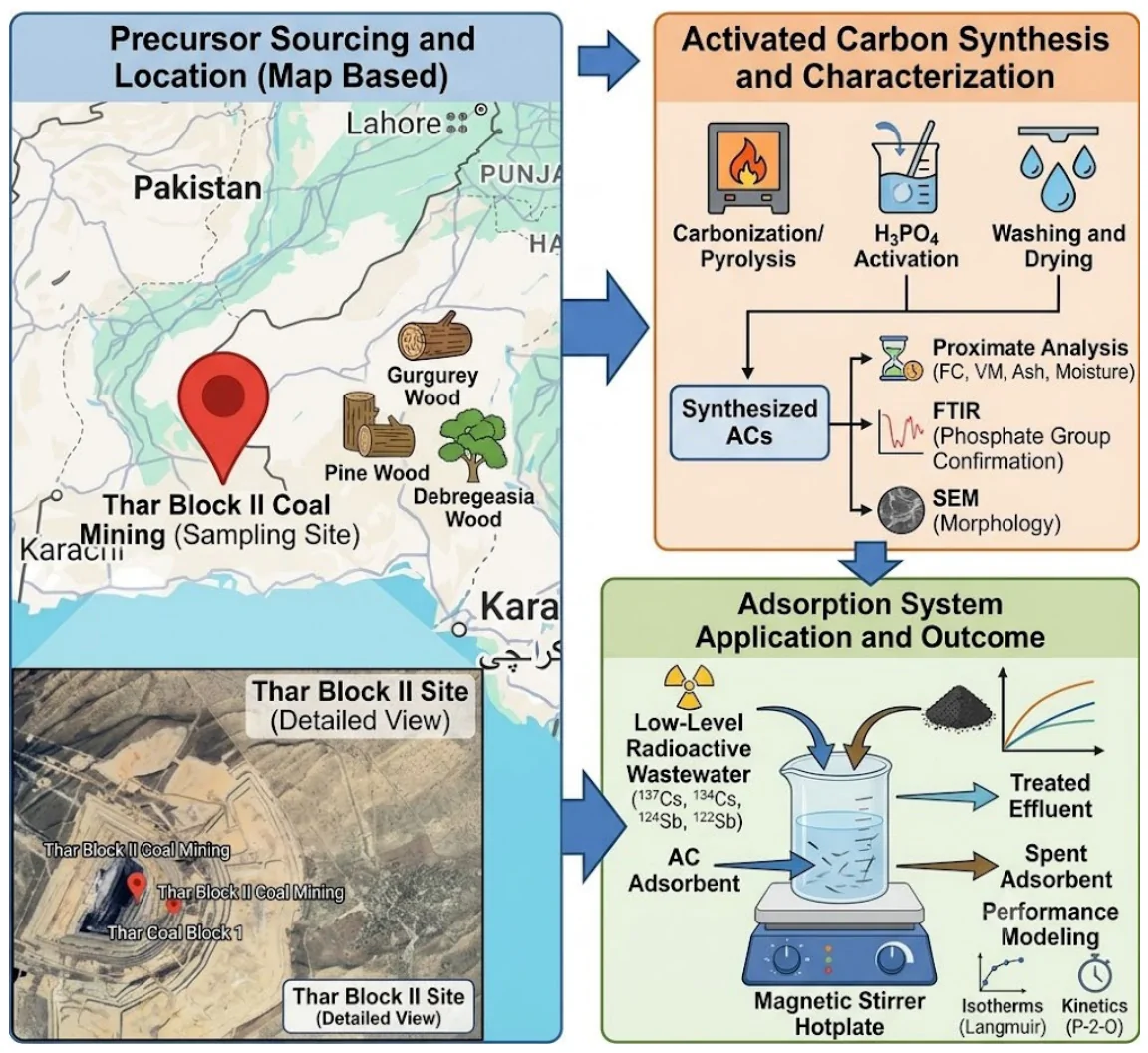
Comprehensive system workflow for multi-nuclide wastewater remediation. Figure 1 is an author-designed schematic illustration. Artificial-intelligence-assisted image generation/editing tools were used only for graphical visualization and layout improvement. All scientific content, experimental design, interpretation, and final verification were performed by the authors.

Seven chemically optimized activated carbons derived from various biomass (Mango, Mesquite, Bhimal, Willow, Debregeasia, Pine, and Gurgurey wood) and Thar coal were used in the present study [32]. The geographical origin and localized satellite view of the source site for the Thar coal sample collected from Tharparkar, Sindh, are depicted in Figure 1. These activated carbons were chosen based on their demonstrated effectiveness in adsorption applications from the cited study. The other materials used were of analytical grade (AG). The schematic (Figure 1) maps out the integration of regional precursors (Thar Block II coal, Gurgurey wood, Pine wood, and Debregeasia wood) from sampling sites, their synthesis pathway via phosphoric acid ($H_{3}{\mathrm{PO}}_{4}$) activation and carbonization, subsequent physicochemical characterization (proximate analysis, FTIR, and SEM), and their application in batch adsorption configurations for the simultaneous sequestration of radioisotopes (Cs 137, Cs 134, Sb 124, and Sb 122) alongside performance modeling.

### 2.2. Low-Level Radioactive Waste Water as Adsorbate

Radioactive coolant wastewater was used to determine the extent of removal of radionuclides by activated carbon. The radioactive wastewater dominantly contained ^137^Cs,^134^Cs, ^124^Sb, ^122^Sb, ^133^Ba, and ^110m^Ag. This study focused on ^137^Cs, ^134^Cs, ^124^Sb, and ^122^Sb removal. The pH of the raw sample was 9–10, while TDS was 1000 mg/L. The initial activity of the radionuclides was 92.3, 30.2, 95.8, and 5.8 Bq/mL of ^137^Cs, ^134^Cs, ^124^Sb and ^122^Sb, respectively.

### 2.3. Proximate Analysis Method

**Figure 2:**
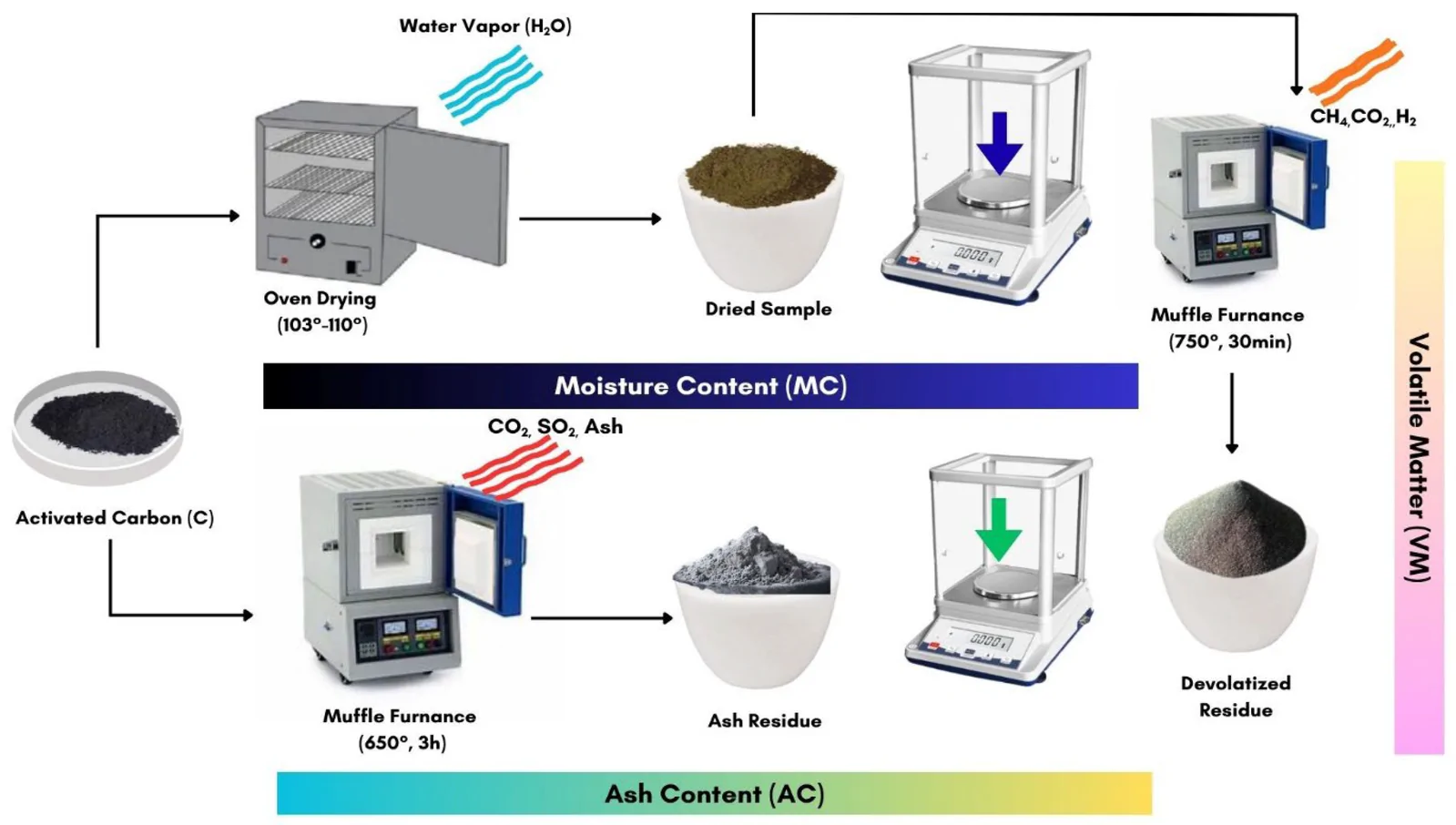
Methodological schematic of the proximate analysis workflow for the synthesized activated carbons. The operational steps illustrate the sequential determination of moisture content (MC) via oven drying at 103–110 °C, volatile matter (VM) fraction through high-temperature de-volatilization in a muffle furnace at 750 °C for 30 min, and ash content (AC) residual determination via complete combustion in a muffle furnace at 650 °C for 3 h.

Proximate analysis was performed according to the American Standards of Testing Materials (ASTM) standards for activated carbon. ASTM D2867-17 is used to determine moisture content, ASTM D5832-98 (2021) for volatile matter, and ASTM D2866-11 for ash content. Fixed carbon is calculated by mass difference [33]. The detailed methodology of the proximate analysis is presented in Section S1. The specific role of Figure 2 is to outline the systematic thermal degradation pathways utilized to quantify the fundamental proximate properties of the synthesized activated carbons. As illustrated in the workflow, targeted moisture evaporation, devolatilization, and ash quantification directly dictate the carbonaceous quality of the materials. The data derived from this matrix validation framework underscores why Gurgurey wood, Pine wood, and Thar coal demonstrated superior matrices, yielding high fixed carbon values (84.3%, 79.5%, and 77.8%) and strictly minimized ash content (3.7%, 5.2%, and 11.47%). Conversely, the processing route defined in Figure 2 highlights the structural limitations of Debregeasia wood, where incomplete carbonization left a high volatile matter fraction (47.3%) and elevated ash residues (18.8%), leaving only 33.9% fixed carbon behind. This structural diagnostic serves as an essential baseline for predicting subsequent multi-nuclide interactions.

### 2.4. Batch Sorption Study

**Figure 3:**
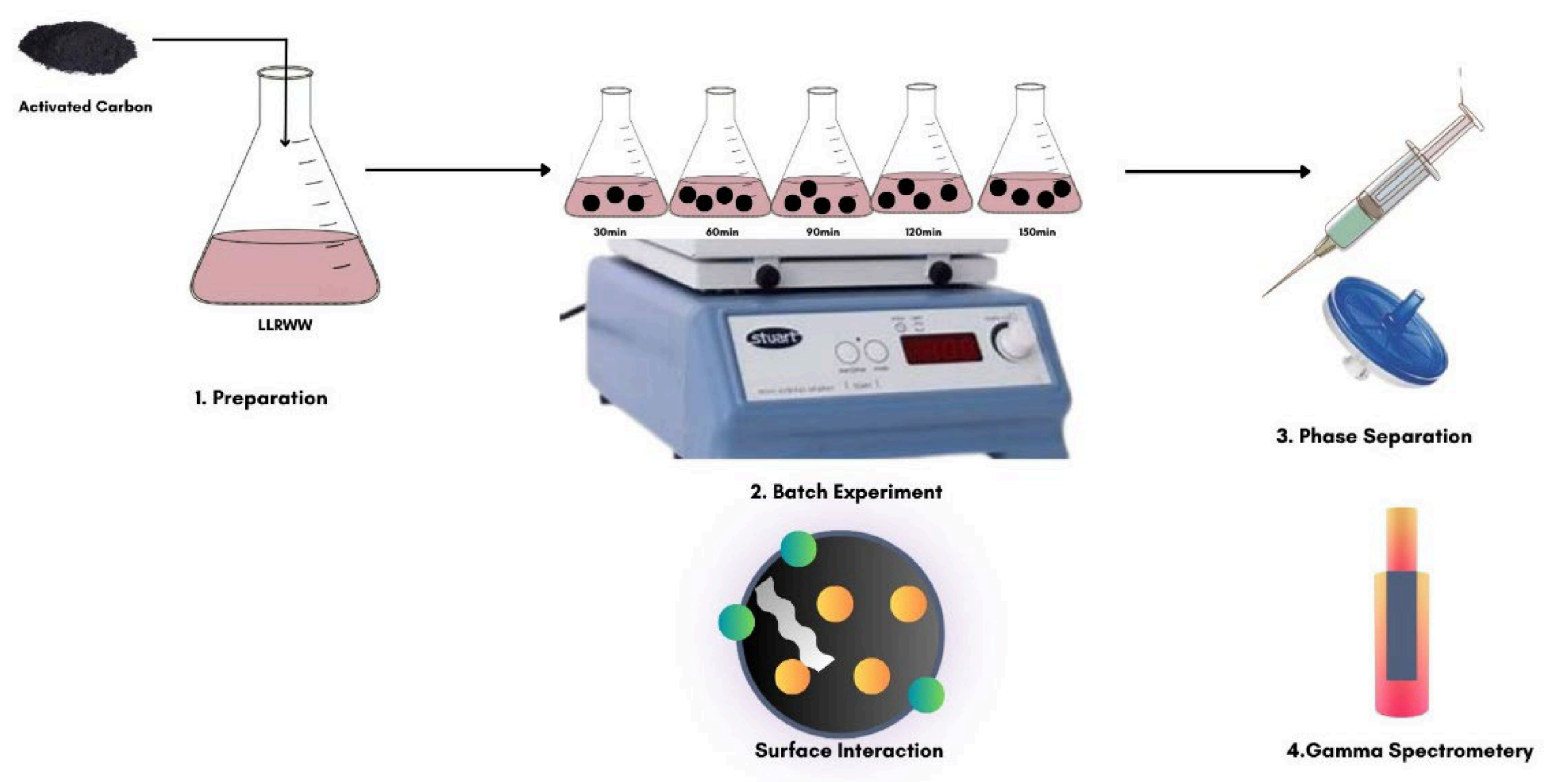
Experimental workflow for the multi-nuclide batch adsorption process. The diagram details the four core stages: (1) preparation of the reaction mixture pairing the activated carbon adsorbent with low-level radioactive wastewater (LLRWW), (2) the batch kinetic experiment conducted on an orbital shaker/stirrer across specific temporal intervals (30 to 150 min), (3) syringe-filter phase separation isolating the spent carbon following surface interactions, and (4) final quantitative analysis via gamma spectrometry.

Batch experiments were performed to check the adsorption efficiency of activated carbons for radionuclides ^137^Cs, ^134^Cs, ^124^Sb, and ^122^Sb from low-level radioactive waste water (LLRWW). The experiments were conducted under normal room conditions (25 °C) at 250 rpm using 10 mL of adsorbate to ascertain the effect of contact time (30, 60, 90, 120, and 150 min) and specific activity (30.2, 60.4, and 90.6 Bq/mL) on the adsorption process. The sorption capacity (q_e_) was calculated using Equation (1):

(1) $$Sorption\;capacity\left(q_{e}\right)=(C_{0}-C_{e})\times \frac{V}{W}$$

where q_e_ (Bq/g) is the uptake in equilibrium. C_e_ (Bq/mL) is the specific activity in equilibrium, and C_0_ (Bq/mL) is the initial adsorbate specific activity, respectively. W (g) is the mass of the adsorbent employed, and V (L) is the volume of the solution. The nature and extent of adsorption were assessed by applying linear and nonlinear isotherm models and kinetics. Details of the kinetic and isotherm models are in Sections S2 and S3 [34]. The step-by-step process of the sorption study is shown in Figure 3. Figure 3 illustrates the comprehensive multi-step radio-analytical workflow executed to monitor the real-time sequestration of the target isotopes. The procedure establishes a controlled boundary condition where fixed mass fractions of the respective activated carbons are placed in contact with the low-level radioactive wastewater containing Cs 137, Cs 134, Sb 124, and Sb 122. The temporal profiling step (30 min to 150 min) directly mapped the chemical uptake rate, allowing accurate determination of the pseudo-second-order kinetic constants. Following the syringe-filter phase separation step shown in Figure 3, the clear liquid effluent was subjected to high-resolution gamma spectrometry to measure the remaining activity. The ultimate success of this batch workflow is reflected in the final data, showing that the high fixed-carbon frameworks mapped in Figure 2 achieved exceptional maximum adsorption capacities (205.6 to 251.9 Bq/g), while the unstable Debregeasia framework dropped as low as 53.4 Bq/g.

### 2.5. Instrumentation

Analysis of the batch experiment was conducted using a Gamma Spectrometry Model GC 3020 manufactured by Canberra Industries (Meriden, CT, USA), based on an evaluation of gamma line energies and peak areas of the gamma rays emitted by radionuclides. The types of functional groups present in activated samples were determined using a Thermo Scientific Nicolet iS50 Spectrometer with the mode version ATR (model number iS50) obtained from Thermo Fisher Scientific, Madison, WI, USA.

## 3. Results and Discussion

### 3.1. Proximate Analysis

A comparative analysis was made between precursors (raw materials) and their resultant activated carbons based on proximate analysis (MC, VM, AC, FC).

#### 3.1.1. Moisture Content (MC)

The charcoals produced from the specified types of woods, namely Mesquite, Pine, Gurgurey, Mango, Bhimal, and Debregeasia wood, had moisture contents (MCs) of 3.4%, 4.8%, 5.4%, 6.5%, 7.8%, and 15.3%, respectively, revealing an increasing trend: Mesquite wood < Pine wood < Gurgurey wood < Mango wood < Bhimal wood < Debregeasia wood [35]. Different studies have measured moisture content within this range, which include *Garcinia schomburgkiana* (7.8%), *Vitellaria paradoxum* (8.40%), *Hevea brasiliensis* (3.26%), *Funtumia elastica* (4.40%), *Prosopis juliflora* (3%), and *Erythrophleum ivorense* (6.28%) [36,37,38,39]. This consistency of the data in the literature further supports the observed moisture content of the wood charcoals in this research. Concerning Thar coal, it contained more moisture, having a value of 43.3%. This result was further proved by the study of Butt et al., in which they found higher moisture content that varied between 42.0% and 50.62% [40]. Similar values for moisture content in Thar coal were also estimated in a study by Choudhary et al. [41]. The presence of high moisture content could be attributed to the presence of aquifers at Thar, Sindh, Pakistan [42].

The moisture content of activated carbons derived from charcoal showed the following trend. Debregeasia wood (12.1%) > Bhimal wood (6.2%) > Mango wood (5.3%) > Mesquite wood (2.4%) > Pine wood (3.8%) > Gurgurey wood (4.2%). In addition, activated carbon obtained from Thar coal had a very low moisture level that could increase to 4.8%. Its percentages suggest the unique porosity and hygroscopic nature of the herbaceous pattern, depicting preliminary moisture retention [43]. Moreover, Thar charcoal exhibited a relatively high moisture content (43.3%) due to its spatial location near aquifers.

The significant reduction in moisture content has been observed after chemical reduction with H_3_PO_4_, which can be justified by an acid-catalyzed dehydration mechanism [44]. H_3_PO_4_ acts as a dehydration agent that facilitates the decomposition of cellulose and hemicellulose in the botanical matrix [45]. This dehydration results in bond breakage of C-O-C and C-O-C, giving water vapor, and constitutes polyphosphate and phosphate bridges that interlink across the carbonaceous template [46]. This structural reduction makes the material more resistant to thermal activity and gives out water molecules [47].

Looking at it from a practical perspective, lower moisture content facilitates the adsorption of radionuclides (^137^Cs, ^122^Sb). Conversely, if the moisture content is high, it encourages water cluster formation, which is produced by hydrogen bonds with oxygen-containing functional groups [48]. Due to steric hindrance and the blockage of pores caused by these clusters, there is a substantial peak in radionuclide adsorption through the internal pore matrix [49]. Moreover, reduced MC ensures increased hydrophobicity, which decreases the competition between water molecules and targeted pollutants for adsorption [50]. Hence, there is an increased surface area for physio-sorption and ion exchange. Table S1 summarizes the moisture content of charcoal and activated carbon.

#### 3.1.2. Volatile Matter

The findings showed that the volatile matter content was 25.8%, 42.3%, 48.5%, 53.6%, 25.2%, and 39.4% for charcoals produced from Mango wood, Mesquite wood, Bhimal wood, Debregeasia wood, Pine wood, and Gurgurey wood, respectively, as presented in Table S1. The volatile matter of charcoals followed a decreasing order: Debregeasia wood > Bhimal wood > Mesquite wood > Gurgurey wood > Mango wood/Pine wood. In earlier studies, Pine wood charcoal had a volatile matter of approximately 42 percent, while for Eucalyptus spp., charcoal was between 25% and 27%, and Mangrove wood charcoal was up to 19.3% [51,52,53].

The findings depicted the following decreasing trend in VM in charcoals: Debregeasia wood (53.6%) > Bhimal wood (48.5%) > Mesquite wood (42.3%) > Gurgurey wood (39.4%) > Mango wood (25.8%) > Pine wood (25.2%). Other research studies have shown VM values for Pine and Eucalyptus charcoals as 42% and 19.3%, respectively. The volatile matter content of Thar coal was also exceptionally high, averaging up to 56.3%, exceeding that of charcoal. This is higher than the reported volatile matter range of 23.9% to 46.88% in Thar coal [42]. The activated carbons synthesized from charcoals showed the following order for VM: Debregeasia wood (47.3%)> Bhimal wood (40.2%) > Mesquite wood (35.6%) > Mango wood (21.2%) > Pine wood (15.3%) > Gurgurey wood (12%). Thar coal possessed the lowest volatile content among activated carbons, i.e., 10.7%. The variation in volatile matter among the plant species is due to their intrinsic lignin-to-cellulose ratio [54]. These lignocellulose biopolymers undergo decomposition at distinctive thermal points [55]. In the temperature range between 220 °C and 400 °C, cellulose and hemicellulose molecules undergo pyrolytic breakage, whereas lignin shows degradation between 100 and 900 °C [56]. During carbonization, the cellulosic, lignin, and hemicellulose components decompose, releasing non-carbon elements such as hydrogen, oxygen, and nitrogen as gases or liquids while leaving behind carbon-rich aromatic structures [57]. The volatile matter level in activated carbon proved to be less than that of charcoal for this reason. However, with regard to volatile matter, the analysis revealed that most of the samples contained the lowest content, with Gurgurey wood measuring only 12%.

The higher volatile matter content in raw Thar coal (56.3%) could have resulted from the oxides present in it. Oxides such as aluminum oxide, silicon dioxide, low ferric oxide, and calcium oxide, with a minor amount of sulfur oxide and manganese oxide, are present in Thar coal [41]. The presence of these unstable oxides resulted in a higher volatile content in raw Thar coal. However, the lower volatile matter in its activated carbon is due to the fact that phosphoric acid impregnation followed by carbonization at 500 °C decomposed the volatile compounds and resulted in their release from coal [58].

#### 3.1.3. Ash Content

Employing the ASTM D2866-11 standard method [59] in the assessment of ash content in charcoals, the ash content was estimated to be 3.8%, 4.6%, 5.4%, 10.6%, 3.3%, and 2.1% for Mango wood, Mesquite wood, Bhimal wood, Debregeasia wood, Pine wood, and Gurgurey wood. After activation, the ash content in these materials rose to 6.8%, 8.3%, 10.1%, 13.5%, 5.2%, and 3.7%, respectively (Table S1). Notably, the activated carbon obtained from Gurgurey wood was found to contain a comparably lower ash content of 3.7%, which falls within the recommended range of 0.5% to 5% for high-quality charcoal [60].

However, other samples of activated carbon derived from various charcoals had a higher ash content percentage in this range. This difference is an indication of the heterogeneity of charcoals and the importance that should be given to the selection of the right raw materials for a certain use. Moreover, a study found ash content in wood charcoals of *Cratoxylum cochinchinense*, *Pettophorum dasylachis*, *Pterocarpus marcocapus*, *Pinus merkusii*, *Sindora siamensis*, and *Keteleeria davidiana* varying from 1.15% to 3.47% [61]. On the other hand, the initial ash content of Thar coal was calculated as 4.09% [40]. Nonetheless, upon activation, the ash content increased to 11.47%. Similar to other reports [62], the ash content of lignite coal (Yallourn coal) also increased up to 12.6% after activation.

This progressive rise in ash content for all samples on activation implies that activation increased the concentration of the mineral residuals. As indicated in Table S1, the ash content of the derived activated carbons was higher than that of the raw material. The derived activated carbons have a higher ash content than precursors. This trend also supports prior studies, whereby there is a direct correlation between a reduction in volatile matter and an increase in ash content [63]. Ash content in activated carbon is regarded as an undesirable element because it reduces its mechanical properties [64,65]. High ash content also affects the adsorption characteristics of activated carbon [66]. Therefore, Debregeasia wood, which contained a higher ash content (13.5%) than the other derived activated carbons, was deemed less desirable for adsorption purposes. Looking purely at ash content, Gurgurey wood was considered more suitable because it contained fewer impurities (3.7%).

#### 3.1.4. Fixed Carbon (FC)

The findings depicted in Table S1 showed that the charcoals from Debregeasia wood, Bhimal wood, Mesquite wood, Gurgurey wood, Mango wood, and Pine wood had fixed carbon contents of 32.9%, 46.1%, 53.1%, 58.5%, 70.4%, and 71.5%, respectively, indicating an increasing trend of Debregeasia < Bhimal < Mesquite < Gurgurey < Mango < Pine wood. In their derived activated carbons, the fixed carbon content also increased in the same order: Debregeasia wood (33.9%), Bhimal wood (49.7%), Mesquite wood (56.1%), Mango wood (72%), Pine wood (79.5%), and Gurgurey wood (84.3%). In recently conducted research, the fixed carbon content of different types of charcoals that were obtained from wood ranged from 76.83% to 90.57% [37].

The rise in the fixed carbon content of activated carbons was due to a decrease in volatile matter. A study also identified an inverse relationship between volatile matter and fixed carbon content [67]. Therefore, the order of fixed carbon content was found to be in the reverse order for volatiles of activated carbons. For volatiles, the decreasing order observed was Debregeasia wood > Bhimal wood > Mesquite wood > Mango wood > Pine wood > Gurgurey wood, while for fixed carbon it was Gurgurey wood > Pine wood > Mango wood > Bhimal wood > Mesquite wood > Debregeasia wood.

The percentage of fixed carbon content in raw Thar coal was initially found to be 39%. This contrasts with the reported figure of 19.6% fixed carbon in Thar coal [68]. However, it is worth mentioning that the value of FC can significantly fluctuate within lignite coals, whereas Simate et al. (2016) described it as ranging from 55 to 70% [69]. After activation, fixed carbon increased up to 77.8%, which is quite high. A possible explanation could be that its impregnation with H_3_PO_4_ and carbonization at 500 °C made chemical transformations in the coal, removing volatile matter and other impurities, which increased its fixed carbon amount. However, as the fixed carbon content rises, an increase in the adsorption capacity of adsorbents was observed [70].

Concluding, maximum fixed carbon was observed in the activated carbon of Gurgurey wood (84.3%), with the least found in Debregeasia-derived activated carbon (33.9%). However, Thar-coal-derived activated carbon had a higher fixed carbon content of up to 77.8%.

### 3.2. FTIR Spectroscopy of Charcoals/Coal-Derived Activated Carbons

The FTIR spectra of activated carbons show the presence of different functional groups, mainly phosphorus–oxygen functional groups and aromatic matrices. For Mango, Bhimal, and Gurgurey, an adsorption spectrum was visible between 1130 and 1151 cm^−1^. In Debregeasia and Gurgurey, an adsorption spectrum was observed between 91 and 74 cm^−1^, which shows the presence of P=O and P-O-C stretches and vibrations in polyphosphate structures. These peaks were sharpest in Debregeasia wood AC because of its high impregnation ratio (1:3). The retention of -OH and -H in Bhimal and Debregeasia at 3100–3700 cm^−1^ affirms that adsorption is facilitated by the complex chemistry formed by phosphoric acid [71].

On the other hand, phosphorus-based functional groups were absent in Pine, Mesquite, and Thar coal. This implies that 500 °C is a high temperature that either destroys the functional groups or evaporates the phosphates for pore formation. The persistent presence of C=C between 1541 and 1614 cm^−1^ confirms a strong carbonaceous framework [72].

**Figure 4:**
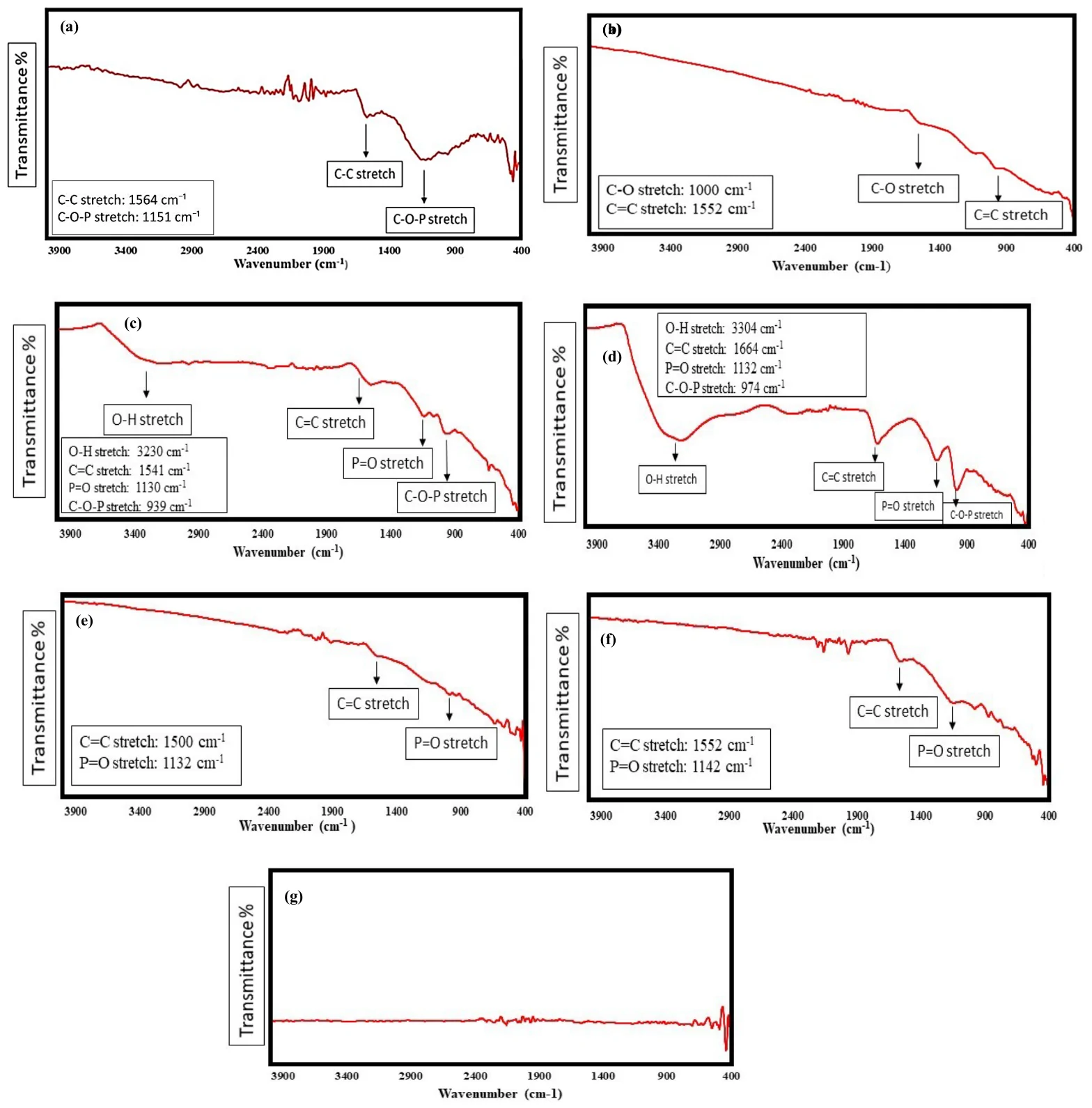
FTIR spectrum of (**a**) Mango, (**b**) Mesquite, (**c**) Bhimal, (**d**) Debregeasia, (**e**) Pine, (**f**) Gurgurey, and (**g**) Thar coal-derived activated carbon without functional groups.

The disappearing C-H aliphatic functional group at 2800–3000 cm^−1^ shows a weak carbonization process and partial removal of volatile matter in Debregeasia AC [73]. The breakage of weak oxygen-containing bonds during pyrolysis is confirmed by weak intensities (in Mesquite and Pine). Conclusively, the FTIR results show that the composition of the parent wood is important while shaping surface functionalization. The recorded vibrational bands and functional profile components across the mapped wave-number range for the various synthesized variants are displayed in Figure 4 (a) Mango, (b) Mesquite, (c) Bhimal, (d) Debregeasia, (e) Pine, (f) Gurgurey, and (g) Thar coal, respectively.

### 3.3. Adsorption Kinetic Models for ^137^Cs, ^134^Cs, ^124^Sb, and ^122^Sb

#### 3.3.1. Adsorption Kinetics of ^137^Cs

**Figure 5:**
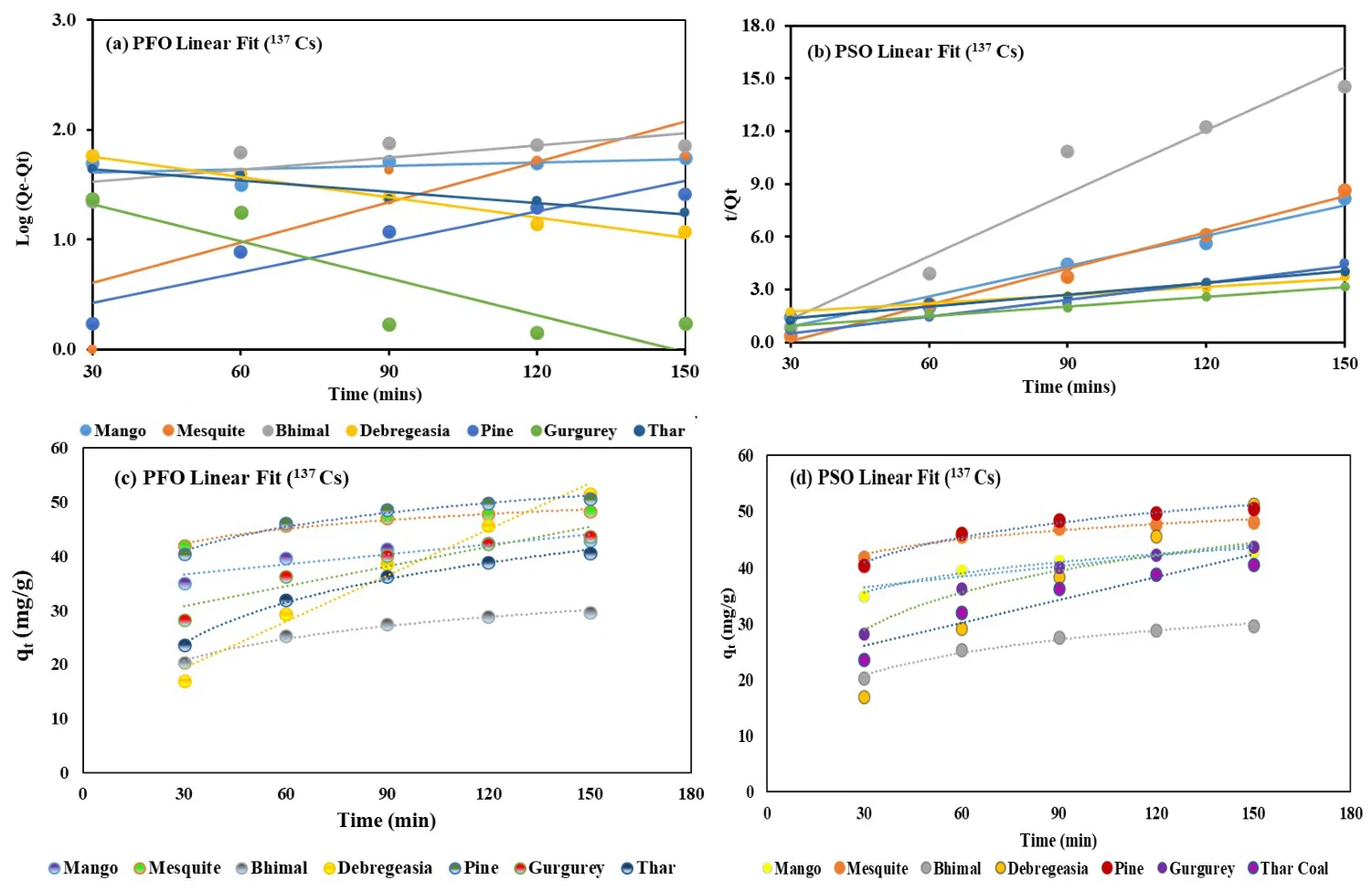
Linear and nonlinear plots for adsorption kinetics of ^137^Cs: (**a**,**c**) pseudo-first-order; (**b**,**d**) pseudo-second-order.

**Figure 6:**
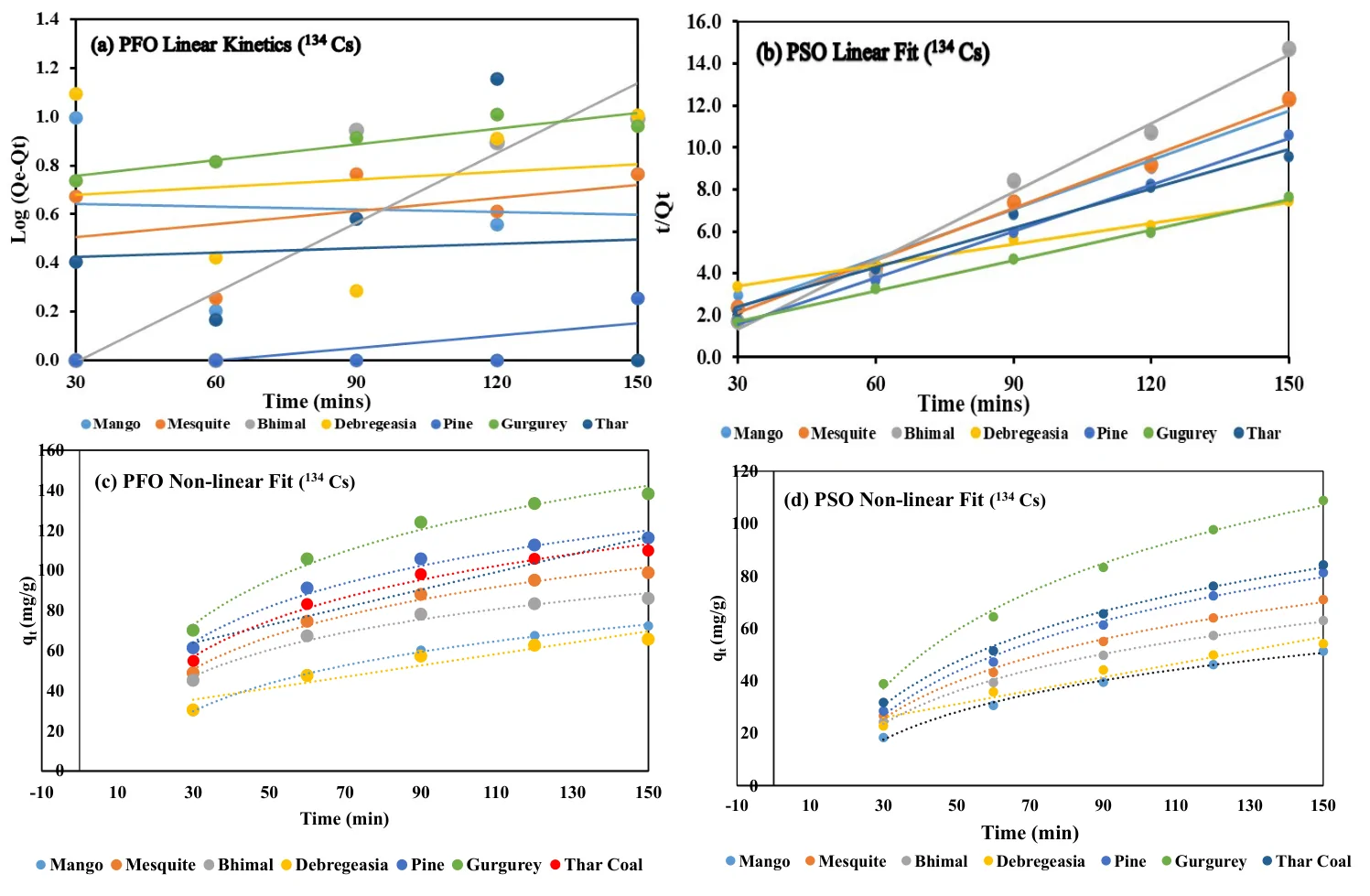
Linear and nonlinear plots for adsorption kinetics of ^134^Cs on activated carbons: (**a**,**c**) pseudo-first-order; (**b**,**d**) pseudo-second-order.

The kinetic study shows that across all activated carbons, the consistent model is pseudo-second order (PSO), as R^2^ = 0.999, and it had lower ARE and SSE (error residuals). The q_e_ experimental (e.g., Pine = 217.4 mg/g) and calculated (e.g., Pine = 211.6 mg/g) values are in closer agreement in PSO compared with PFO [Tables S2–S5]. On the other hand, nonlinear kinetics provided greater statistical validation than linear regression, supporting the prevalence of the PSO framework and suggesting that the adsorption reaction is governed by chemisorption involving ion exchange and surface interactions between activated carbon functional groups and ^137^Cs [74]. The isotherm parameters for ^124,122^Sb are mentioned in Tables S3–S6. Graphs of the kinetics for ^137^Cs and ^134^Cs are represented in Figure 5 and Figure 6.

#### 3.3.2. Adsorption Kinetics of ^134^Cs

The most consistent model for adsorption of ^134^Cs is nonlinear pseudo-second-order (PSO) rather than PFO [Table S3], whereas in PFO, q_e_ experimental and calculated values are inconsistent. Nonlinear PSO has an R^2^ value between 0.994 and 0.999, and it has the lowest ARE and SSE values. The rate of reaction is chemisorption, which is inferred on the basis of consistency between q_e_ experimental and q_e_ calculated in nonlinear PSO [75].

The pseudo-second-order kinetic model’s dominance is further supported by the kinetic parameters for ^134^Cs adsorption in Table S6. For this model, all adsorbents exhibit R^2^ values above 0.98, and the computed Q_e_ values closely resemble the experimental ones. For example, Gurgurey carbon had R^2^ = 0.99, a Q_e_ (calc) of 149.3 Bq/g, and a Q_e_ (exp) of 146.2 Bq/g. This pattern implies that the regulating step in ^134^Cs adsorption is chemisorption, most likely involving surface complexation or ion-exchange mechanisms [76]. With low R^2^ values and significant differences between calculated and experimental Q_e_, the pseudo-first-order model performed poorly, confirming that physiosorption is insufficient to describe the adsorption process [77]. The findings show that the superior kinetic performance of coal carbons from Gurgurey, Pine, and Thar is a result of their superior textural and chemical surface properties. The isotherm parameters for ^134^Cs are mentioned in Table S3. Graphs of the kinetics for ^134^Cs are represented in Figure 6.

#### 3.3.3. Adsorption Kinetics of ^124^Sb

Nonlinear PSO shows the highest alignment based on its kinetic parameters. Its R^2^ value is 0.992–0.999, and it has the lowest error percentages. The q_e_ experimental and q_e_ calculated values are consistent for both PFO and PSO. It suggests that the adsorption reaction follows chemisorption. The highest adsorption capacities are shown by Gurgurey (251.9 mg/g) and Pine (229.4 mg/g), proving that the nonlinear model is more valid and reliable for determining kinetic parameters for ^124^Sb [78].

**Figure 7:**
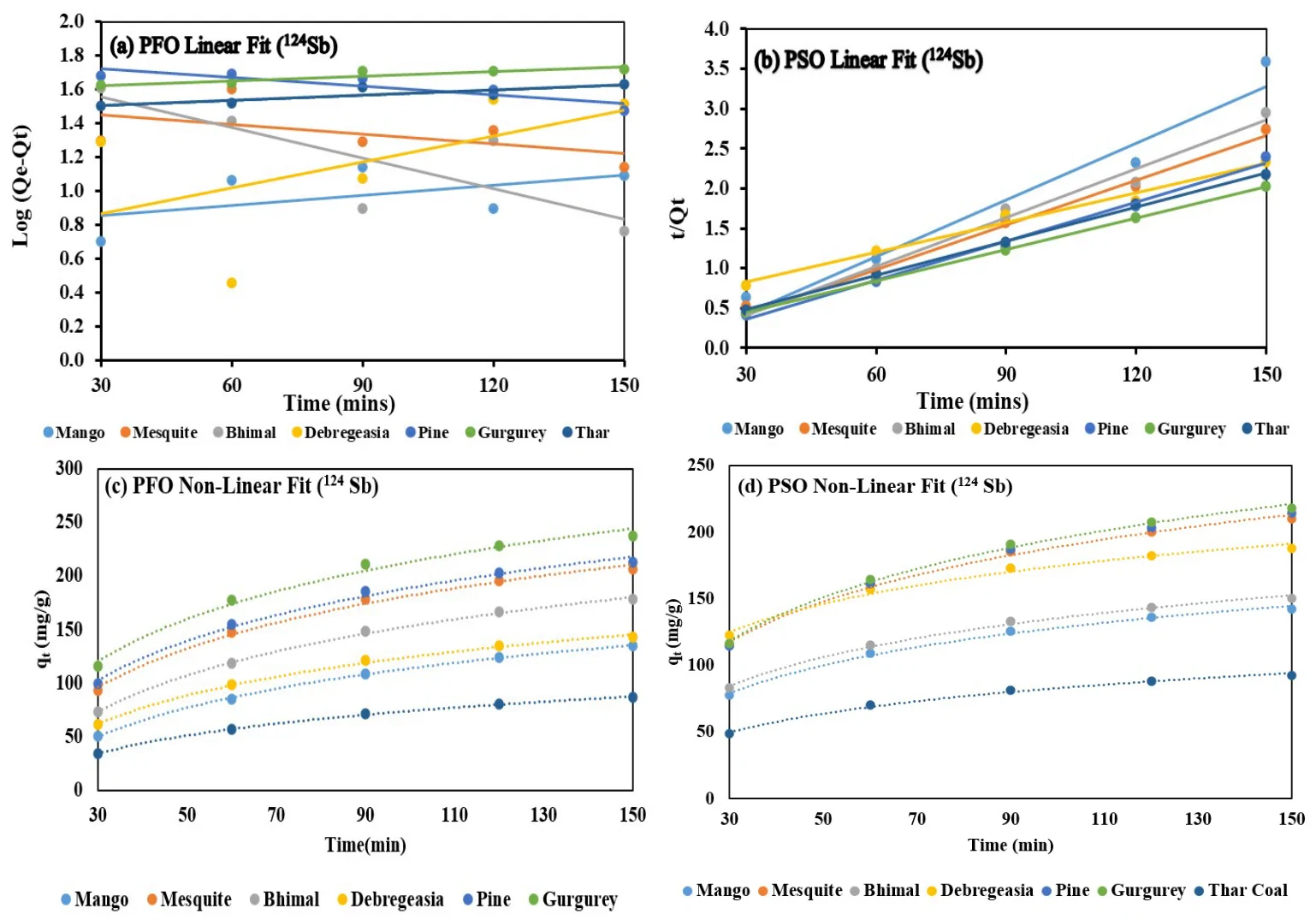
Linear and nonlinear plots for adsorption kinetics of ^124^Sb on activated carbons: (**a**,**c**) pseudo-first-order; (**b**,**d**) pseudo-second-order.

According to Table S4, the pseudo-second-order kinetic model once more provides a better fit to the data than the pseudo-first-order model for ^124^Sb adsorption. The adsorption mechanism is governed by chemisorption, as confirmed by the high R^2^ values (up to 0.99), as well as the slight variations between Q_e_ (calc) and Q_e_ (exp). Pine activated carbon, for instance, displayed Q_e_ (calc) = 232.6 Bq/g and Q_e_ (exp) = 229.4 Bq/g, with R^2^ = 0.99. Faster uptake kinetics were indicated by the higher initial adsorption rate (h) for the more effective adsorbents, such as Pine and Gurgurey. Lower R^2^ and mismatched Q_e_ values show that the pseudo-first-order model failed to adequately describe adsorption behavior [79]. These findings demonstrate how certain activated carbons’ well-developed pore structures and active sites have a major impact on the adsorption rates of ^124^Sb. Graphs of the kinetics for ^124^Sb are represented in Figure 7.

#### 3.3.4. Adsorption Kinetics of ^122^Sb

The adsorption kinetics of ^122^Sb are again consistent with nonlinear PSO, as its R^2^ values are between 0.992 and 0.999 [Tables S7–S9]. On the other hand, PFO depicts inconsistent correlations between the kinetic parameters, whereas the error values of PSO are the lowest. The q_e_ experimental values for Gurgurey (128.2 mg/g) and Thar coal (232.6 mg/g) are consistent with q_e_ calculated values. The results show that Thar coal is the most efficient adsorbent, with an adsorption capacity of 232.3 mg/g dominating the biomass-based activated carbons.

**Figure 8:**
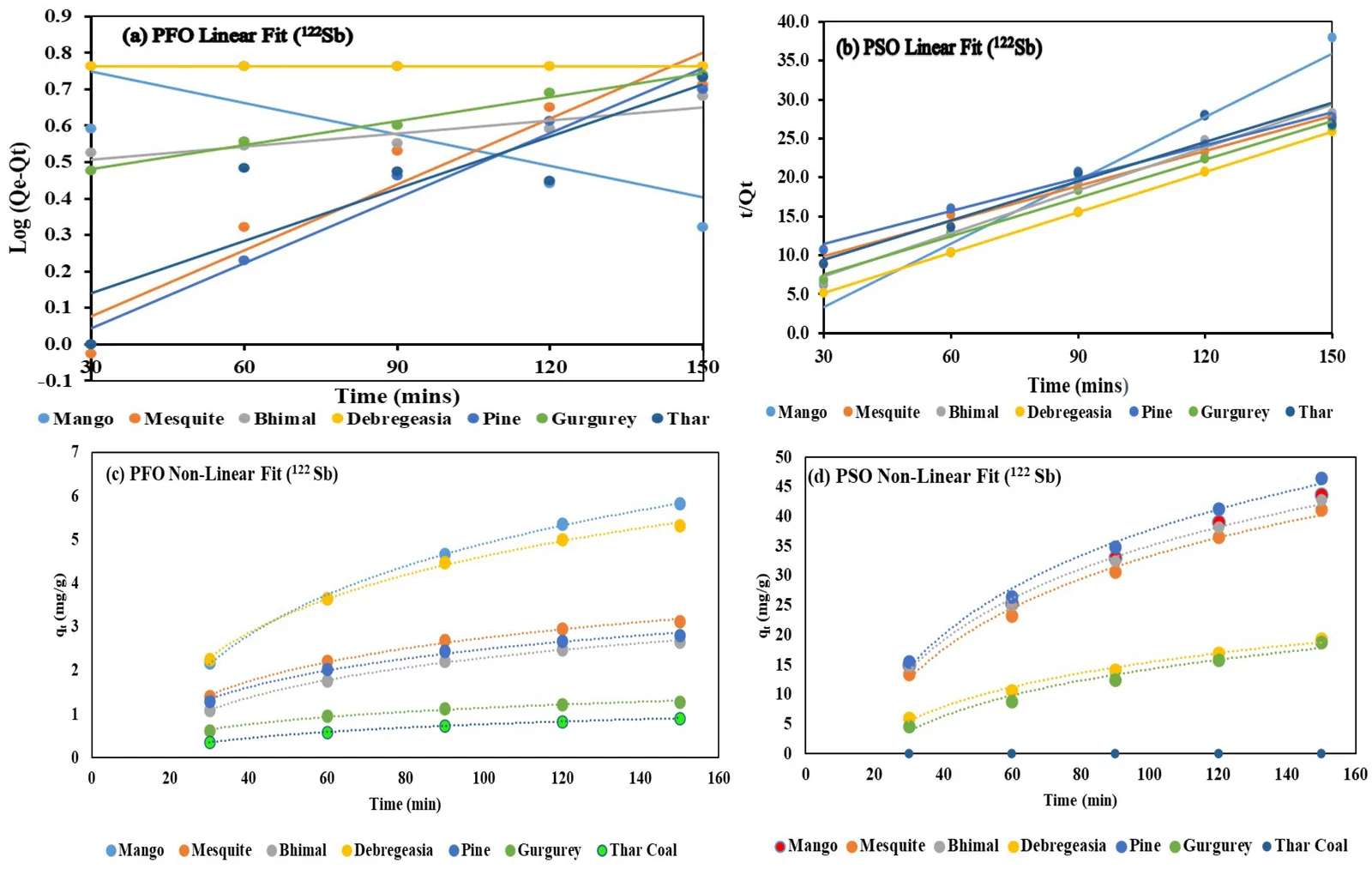
Linear and nonlinear plots for adsorption kinetics of ^122^Sb on activated carbons: (**a**,**c**) pseudo-first-order; (**b**,**d**) pseudo-second-order.

R^2^ values close to or equal to 0.99 for the majority of adsorbents in Table S5 demonstrate a strong preference for the pseudo-second-order model in the adsorption kinetics for ^122^Sb [Tables S7–S9]. For example, the carbon derived from Mesquite wood had Q_e_ (calc) = 95.2 Bq/g and Q_e_ (exp) = 91.9 Bq/g, with R^2^ = 0.989. These results suggest that chemisorption, which involves electron-sharing or surface complexation, is the mechanism by which ^122^Sb adsorption takes place. This is a clear indication that chemisorption was the rate-determining step and the main mechanism through which activated carbons adsorbed ^122^Sb [80]. High adsorption capacities and kinetic compatibility were once again shown by carbons based on Gurgurey and Pine. The pseudo-first-order model appears to be unsuitable for explaining ^122^Sb uptake, as evidenced by its larger discrepancies and lower R^2^ values. The outcomes are in line with previous research, demonstrating that the sorption kinetics are significantly influenced by surface chemistry and fixed carbon content. The kinetic parameters for ^122^Sb are mentioned in Tables S5, S7 and S8. Graphs of the kinetics for ^122^Sb are represented in Figure 8.

### 3.4. Adsorption Isotherm Models for ^137^Cs, ^134^Cs, ^124^Sb, and ^122^Sb

#### 3.4.1. Adsorption Isotherms of ^137^Cs

**Figure 9:**
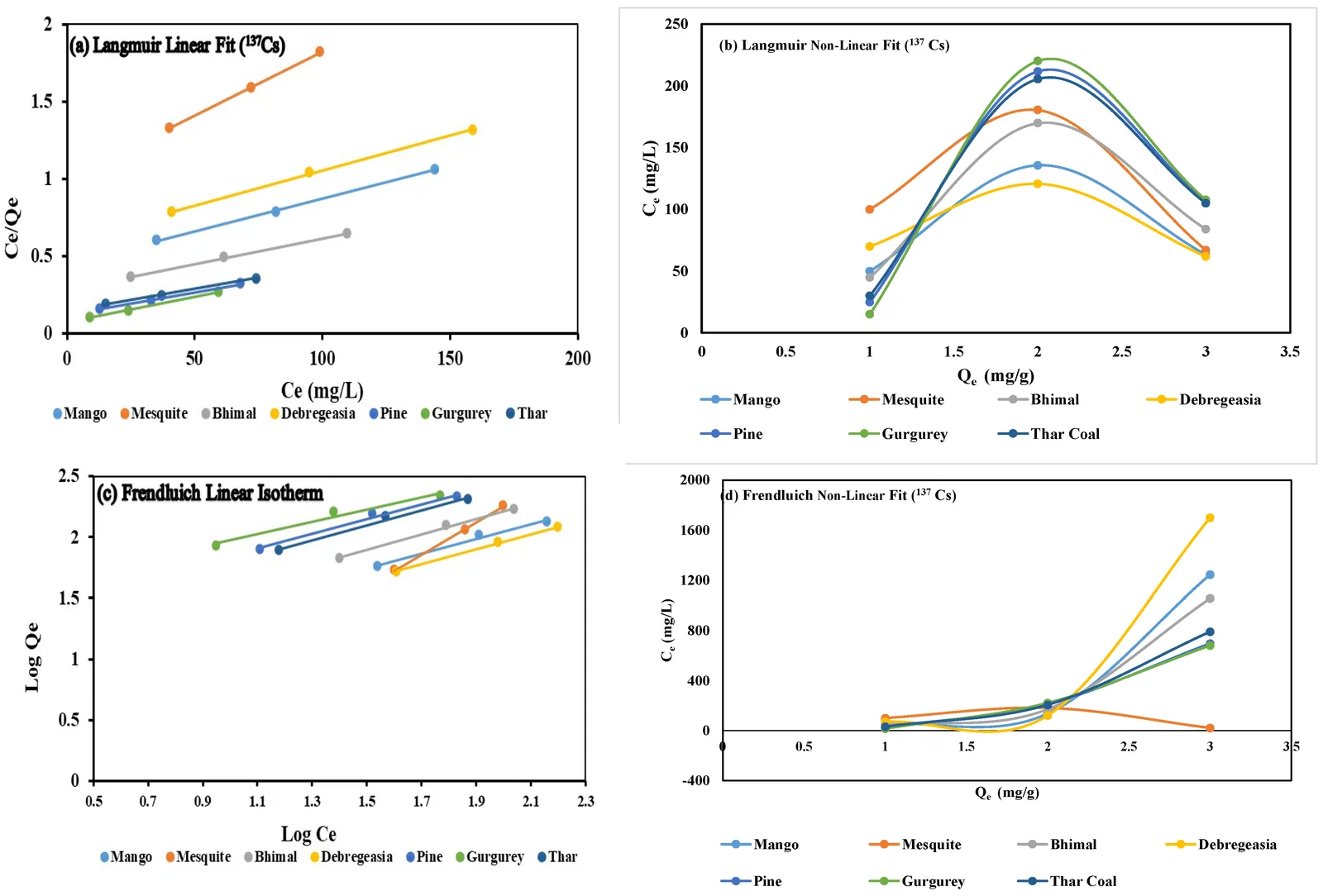
Linear and nonlinear plots for adsorption isotherms of ^137^Cs on activated carbons: (**a**,**b**) Langmuir isotherm; (**c**,**d**) Freundlich isotherm.

The results show that nonlinear Langmuir isotherm model is more consistent with the adsorbents (R^2^ = 0.999) and shows a consistent alignment between experimental and calculated q_e_. This depicts that adsorption is happening in a monolayer with homogenous coverage, where each active site is occupied by one ion at a time [81]. For instance, Gurgurey AC had R^2^ = 0.99, Q_e_ = 220.2 Bq/g, and Q_max_ = 222.2 Bq/g. In every instance, the separation factor (R_L_) values fell between 0 and 1, suggesting that the adsorption was favorable. The slightly lower R^2^ values of the Freundlich model imply that multilayer or heterogeneous adsorption was less dominant, despite the fact that it also exhibits good correlation [82]. The high fixed carbon and developed surface area of adsorbents derived from the coals of Gurgurey, Pine, and Thar are correlated with their strong performance [83]. On the other hand, the Freundlich model has higher correlation constants for Mango and Pine, but these are inconsistent with the calculated yields. The isotherm parameters for ^137^Cs are mentioned in Table S6. The graphs of Langmuir and Frendluich for ^137^Cs are represented in Figure 9.

The Temkin model gives information about adsorbent and pollutant surface interaction and energies. It implies that adsorption heat decreases subsequently due to the formation of a monolayer on the adsorbent surface. The values of the Temkin constant range from 25.4 to 48.3, suggesting that physical-chemical adsorption is the main process for the adsorption mechanism. The slightly lower affinity values for the Temkin constant suggest that surface interaction energy is not the main force for the adsorption process [84]. The parameters for the Temkin model are mentioned in Table S10.

**Figure 10:**
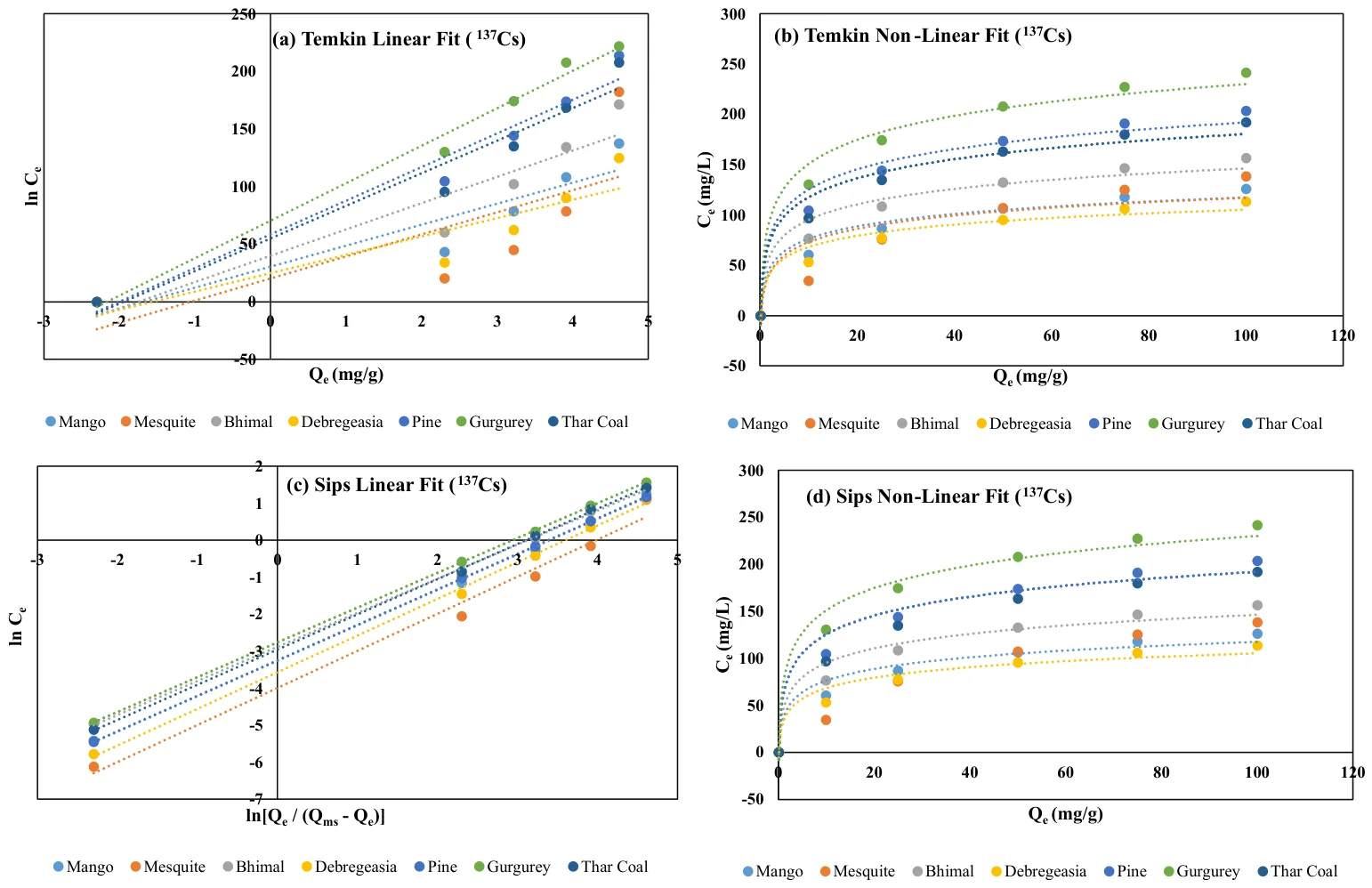
Linear and nonlinear plots for adsorption isotherms of ^137^Cs on activated carbons: (**a**,**b**) Temkin isotherm; (**c**,**d**) Sips isotherm.

The Sips model for ^137^Cs is a mix of Langmuir and Freundlich isotherms. The β values for all adsorbents are close to 1 (0.94–1.13). It suggests that adsorption is in a homogeneous Langmuir monolayer. The maximum adsorption capacities are for Gurgurey (224.8 mg/g) and Pine (214.5 mg/g), making them the most efficient adsorbents for radioactive wastewater treatment. The Sips model fitting indicates that saturation occurs at a higher concentration, which is an important aspect to be considered for industrial wastewater treatment [85]. The parameters for the Sips model are mentioned in Table S10. The graphs of Temkin and Sips for ^137^Cs are represented in Figure 10.

#### 3.4.2. Adsorption Isotherms of ^134^Cs

The isotherm model for ^134^Cs is consistent with both the Langmuir and Freundlich isotherms. However, the values of the parameters show that adsorption is more consistent with the nonlinear Langmuir model (R^2^ = 0.998).

**Figure 11:**
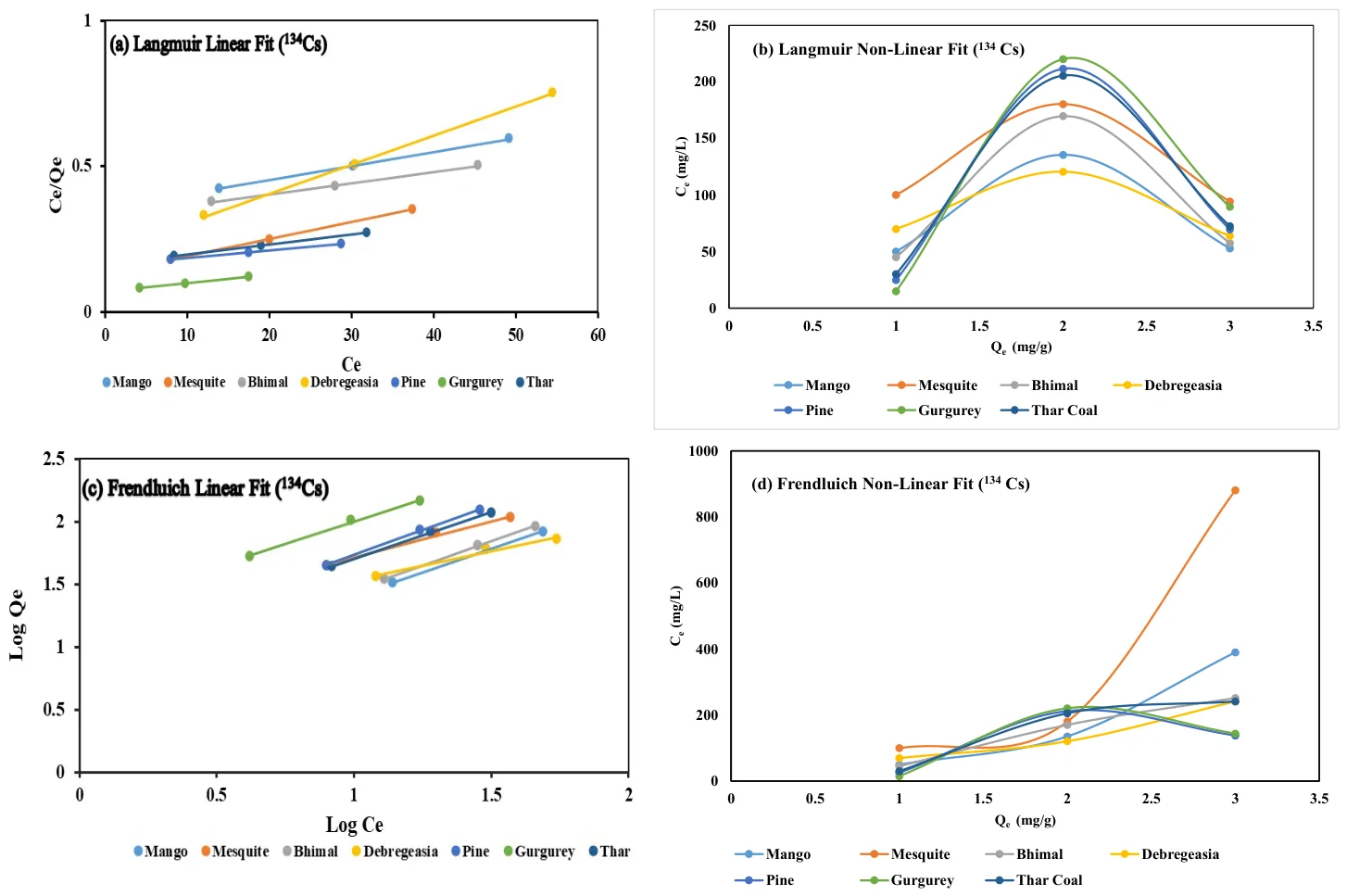
Linear and nonlinear plots for adsorption isotherms of ^134^Cs on activated carbons: (**a**,**b**) Langmuir isotherm; (**c**,**d**) Freundlich isotherm.

The Langmuir model offers the best fit (R^2^ > 0.95 for all adsorbents), as shown by the adsorption isotherms of ^134^Cs in Table S10, which also shows monolayer coverage and uniform adsorption sites [86]. The experimental Q_e_ values and the Langmuir-derived Q_max_ values are in good agreement; for example, Gurgurey AC had Q_e_ = 146.2 Bq/g and Q_max_ = 149.3 Bq/g, with R^2^ = 0.99. The lower R^2^ values and higher 1/n values show a weaker fit than Langmuir, even though Freundlich parameters also point to effective sorption (*n* > 1). The findings confirm that surface saturation is attained during Cs adsorption and that carbon pore structure and surface functionality are important factors in uptake capacity [87]. The isotherm parameters for ^134Cs^ are mentioned in Table S7. The graphs of Langmuir and Frendluich for ^134^Cs are represented in Figure 11.

The Temkin model depicts a sequential decrease in adsorption heat with adsorption coverage, as calculated by B. It implies a physical–chemical adsorption connection between the adsorbent and the pollutant [88]. Gurgurey and Mesquite have high A_T_ (binding coefficient) values. This suggests a strong initial affinity for ^134^Cs. There is a strong correlation present, yet the R^2^ values are low, suggesting that energy forces are present but not enough to define the adsorption process. The parameters for the Temkin model are mentioned in Table S11.

**Figure 12:**
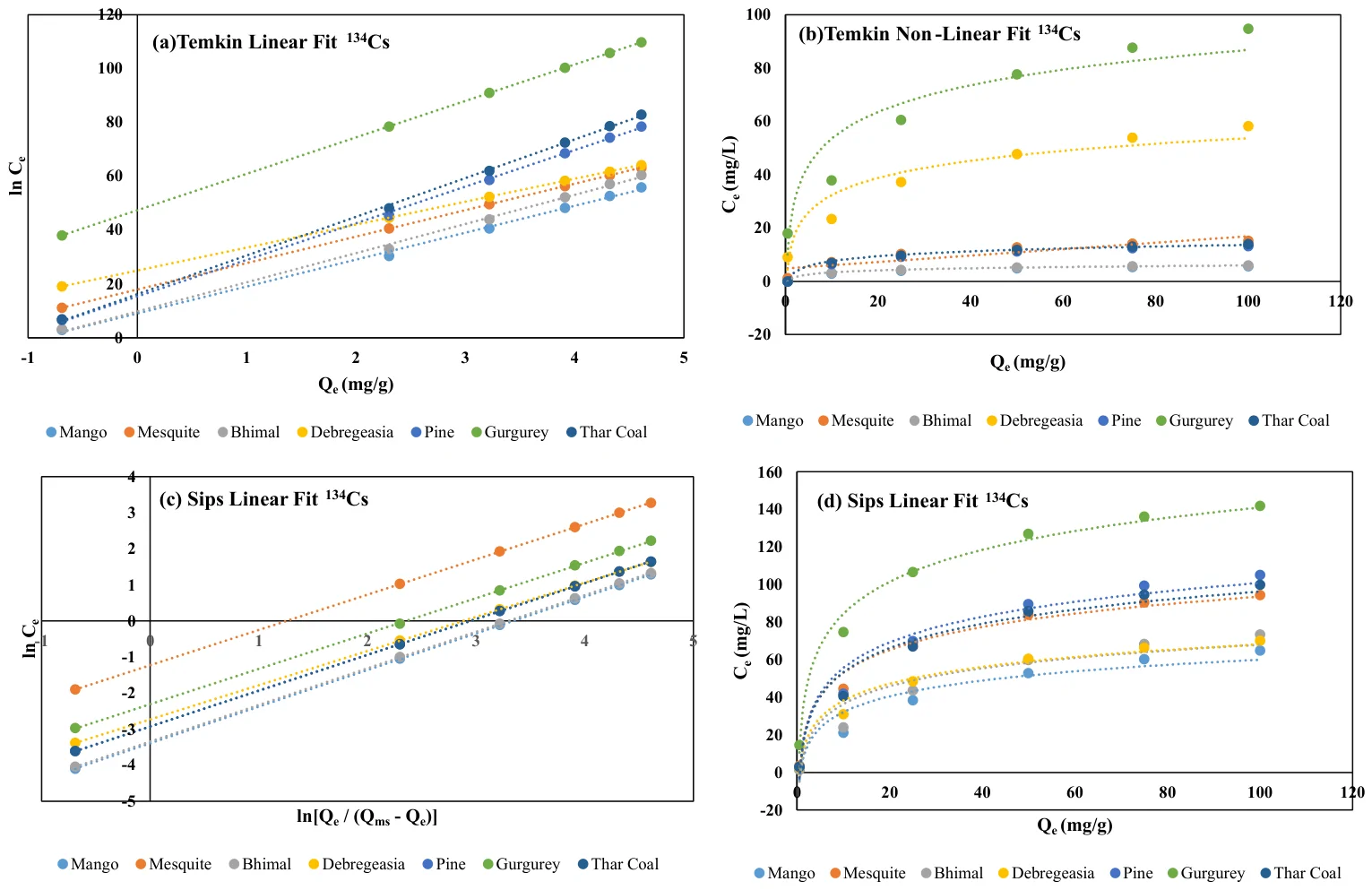
Linear and nonlinear plots for adsorption isotherms of ^134^Cs on activated carbons: (**a**,**b**) Temkin isotherm; (**c**,**d**) Sips isotherm.

The R^2^ values of the Sips model provide mathematical authenticity, as these are close to 0.999 for all adsorbents. The value of β suggests that adsorption-pollutant interactions are strongly monolayer-Langmuir on uniform surfaces [89]. Gurgurey and Pine show the highest capacities (i.e., 149.3 mg/g and 125 mg/g), making them effective adsorbents. The isotherm models suggest that biomass-based adsorbents reach saturation points in a monolayer configuration. The parameters for the Sips model are mentioned in Table S11. The graphs of Temkin and Sips for ^134^Cs are represented in Figure 12.

#### 3.4.3. Adsorption Isotherms of ^124^Sb

**Figure 13:**
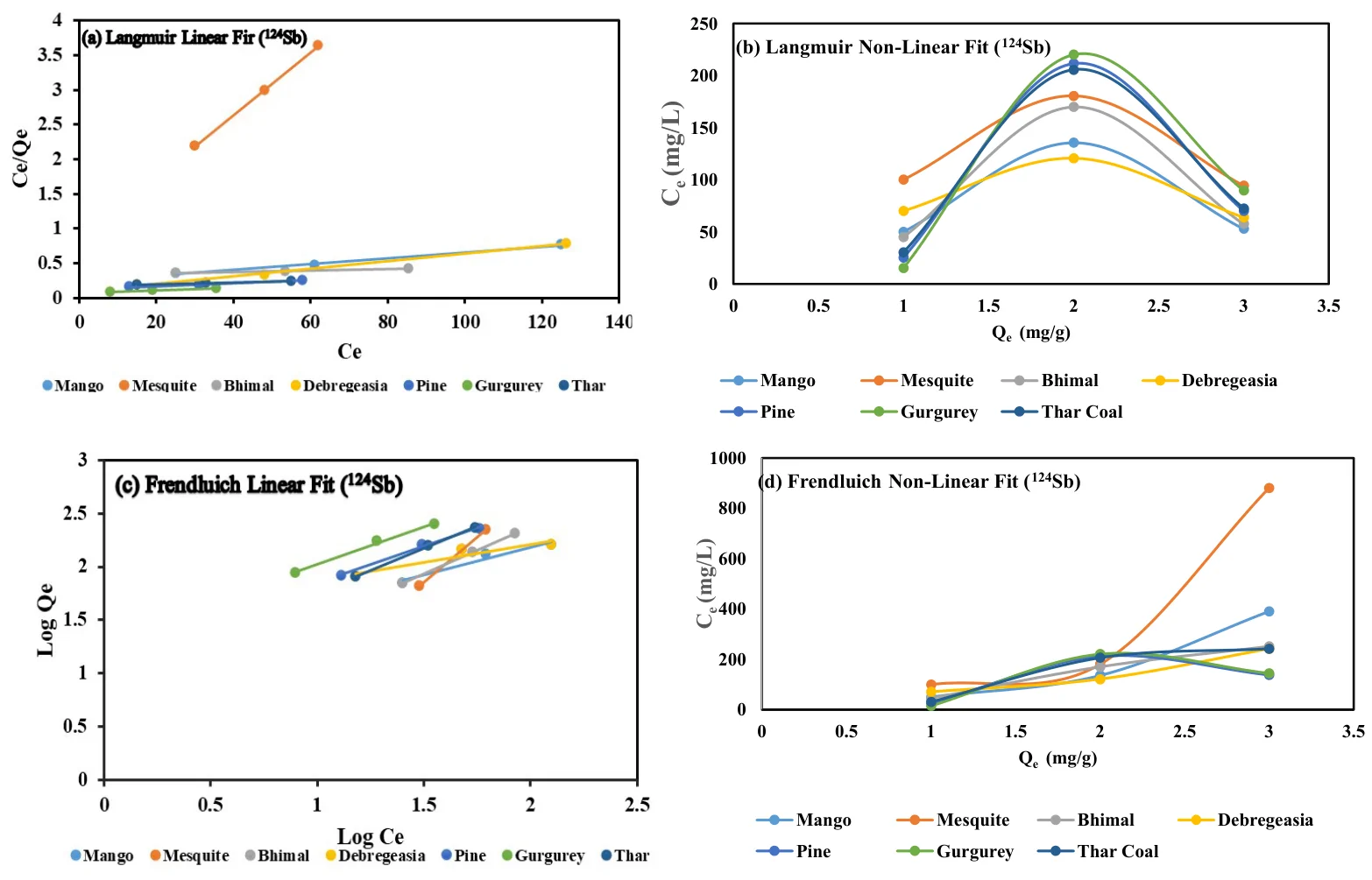
Linear and nonlinear plots for adsorption isotherms of ^124^Sb on activated carbons: (**a**,**b**) Langmuir isotherm; (**c**,**d**) Freundlich isotherm.

The adsorption isotherm results for ^124^Sb are displayed in Table S8, which emphasizes how well the Langmuir model fits all adsorbents. Monolayer adsorption is confirmed as the governing mechanism by the close match between Q_e_ and Q_max_ (e.g., 251.9 Bq/g vs. 256.4 Bq/g for Gurgurey) and the R^2^ values, which consistently reach or exceed 0.97 [90]. Strong affinity and effective adsorption sites are suggested by the Langmuir b values and favorable R_L_ values. The slightly lower correlation coefficients indicate that the Freundlich model is secondary to Langmuir in describing ^124^Sb adsorption, even though it indicates multilayer adsorption because *n* > 1 [91]. All things considered, the evidence points to a strong interaction between ^124^Sb and uniformly distributed functional sites, particularly in well-activated carbons [92]. The graphs of Langmuir and Frendluich for ^124^Sb are represented in Figure 13.

The Temkin isotherm gives information on adsorption heat and surface interaction energy between the adsorbent and the pollutant. The value of B (34.18–69.15) implies physical–chemical adsorption. This shows that adsorption heat successively decreases with an increase in surface coverage [93]. The highest R^2^ values are for Gurgurey and Thar coal, while the lowest are for Mango and Debregeasia (as shown in Table S12). This demonstrates that surface complexity cannot be demonstrated by adsorption heat.

**Figure 14:**
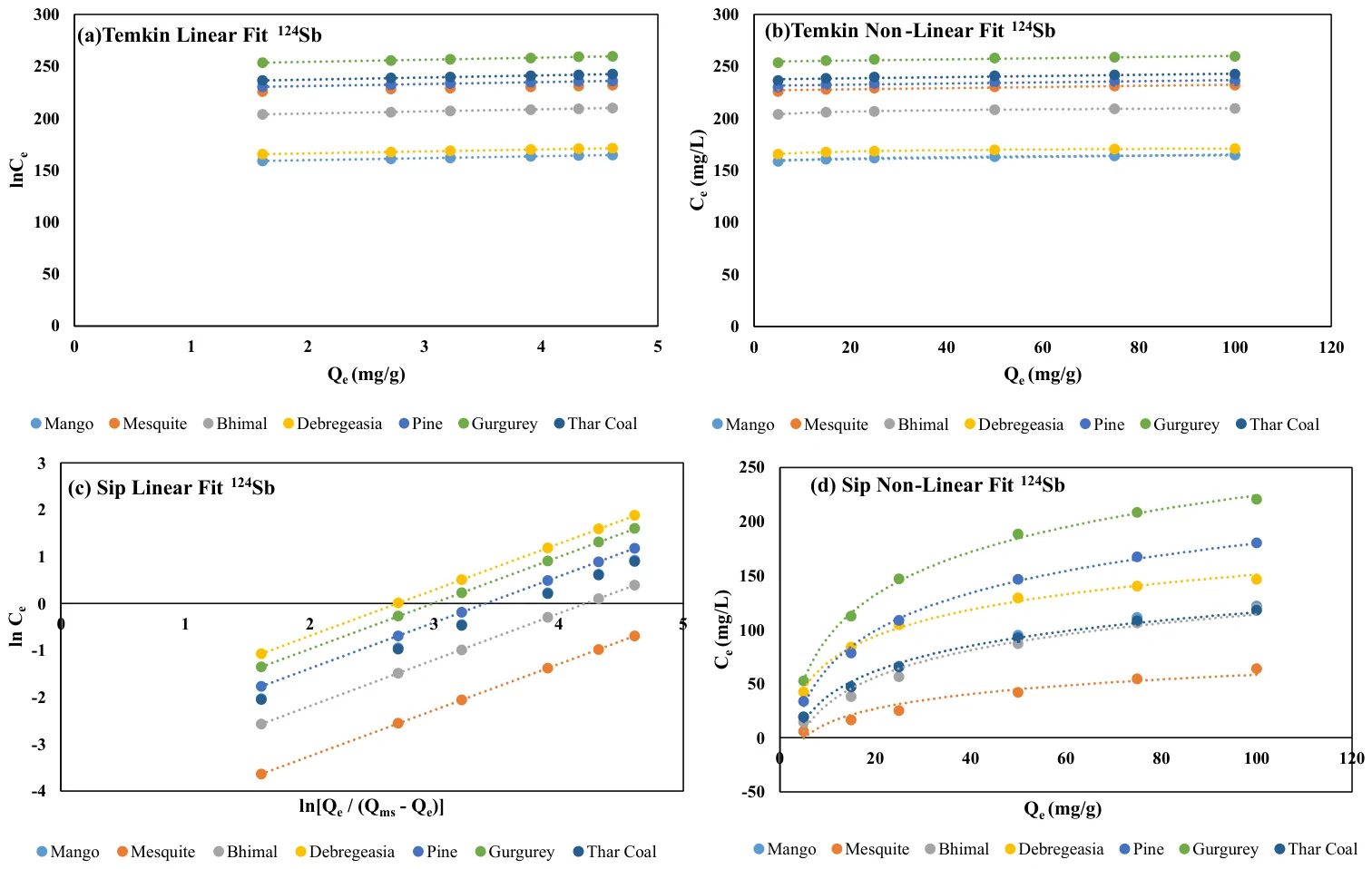
Linear and nonlinear plots for adsorption isotherms of ^124^Sb on activated carbons: (**a**,**b**) Temkin isotherm; (**c**,**d**) Sips isotherm.

The correlation factor for Sips is ideal, as the R^2^ values for all adsorbents are approximately 0.999. β is close to 1, showing that adsorption is in a homogenous Langmuir monolayer. It means that saturation occurs in a single-layer form, and no more adsorbent sticks after one layer [94]. The adsorption capacities of Gurguey (256.4 mg/g) and Thar coal (238.1 mg/g) show the highest removal efficacy for ^124^Sb radioactive wastewater. The parameters for the Sips model are mentioned in Table S12. The graphs of Temkin and Sips for ^124^Sb are represented in Figure 14.

#### 3.4.4. Adsorption Isotherms of ^122^Sb

**Figure 15:**
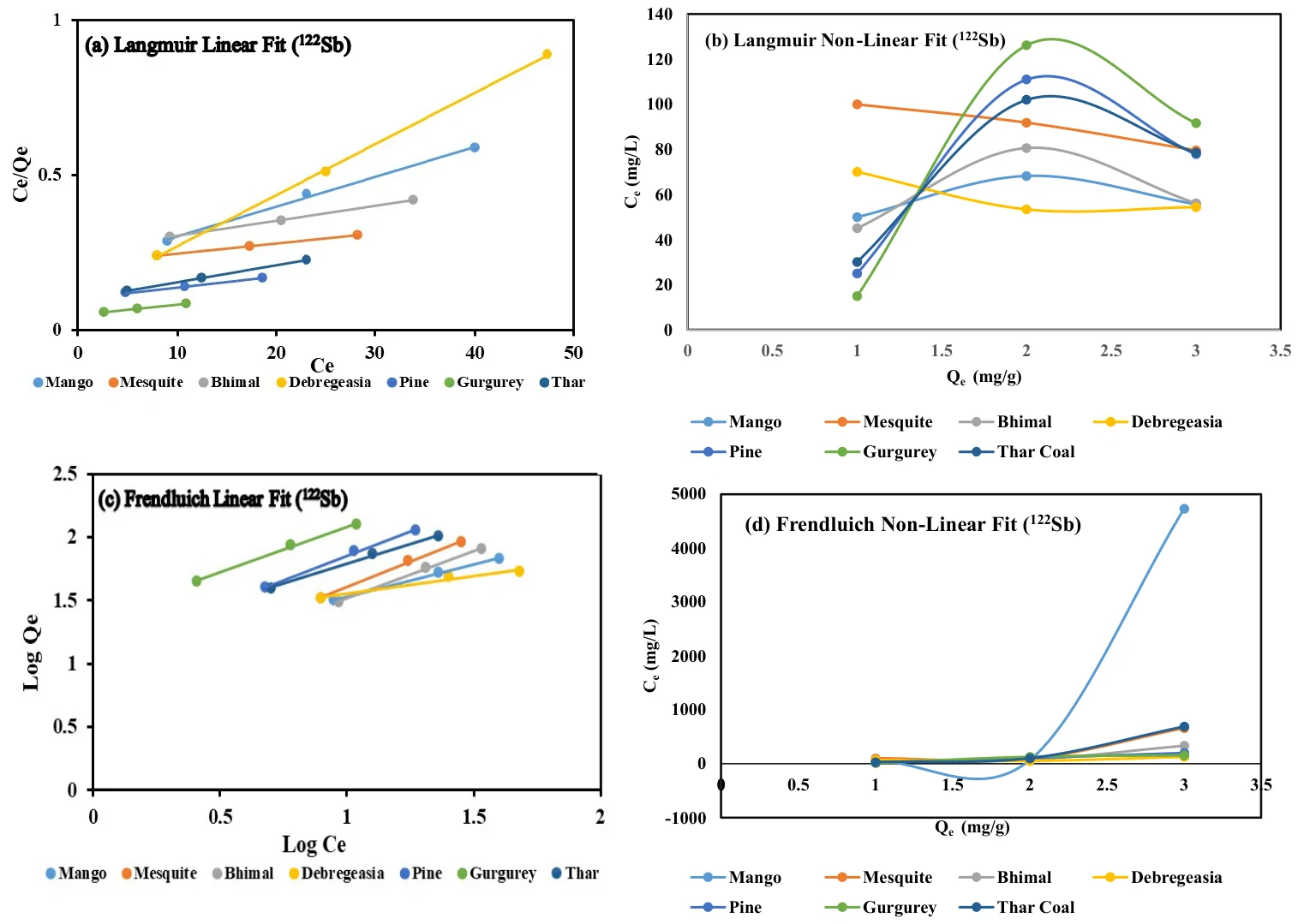
Linear and nonlinear plots for adsorption isotherms of ^122^Sb on activated carbons: (**a**,**b**) Langmuir isotherm; (**c**,**d**) Freundlich isotherm.

The isotherm model for adsorption of ^122^Sb is consistent with the nonlinear Langmuir model (R^2^ = 0.999), suggesting monolayer adsorption on a homogeneous surface. The R_L_ values for all adsorbents are below 1, inferring that adsorption of ^122^Sb is favorable across all botanical matrices [95]. The Q_max_ values closely resemble the experimental adsorption capacities (e.g., Gurgurey AC: Q_e_ = 126.2 Bq/g, Q_max_ = 129.9 Bq/g), and the R^2^ values for Langmuir typically surpass 0.91. These findings support the notion that monolayer adsorption predominates, with favorable R_L_ values indicating efficient uptake. The slightly lower R^2^ values imply that multilayer adsorption is less likely, even though Freundlich parameters show physical adsorption on heterogeneous surfaces (*n* > 1) [96]. Because adsorption is driven by surface homogeneity and functional group interaction, Langmuir’s model more accurately captures the behavior of ^122^Sb. The isotherm parameters for ^122^Sb are mentioned in Table S9. The graphs of Langmuir and Frendluich for ^122^Sb are represented in Figure 15.

The B values for the Temkin model of the isotherm range from 11.55 to 23.75. It demonstrates less adsorption heat, suggesting alignment with physical–chemical adsorption. Debregeasia and Gurgurey have relatively higher A_T_ values, which suggests that they have good and high affinity for ^122^Sb at the beginning of the adsorption coverage [97]. The correlation constant value of Temkin (R^2^ = 0.97) shows homogenous and uniform interactions on the adsorbent surface rather than being dependent on heat distribution [98]. The parameters for the Sips model are mentioned in Table S13.

**Figure 16:**
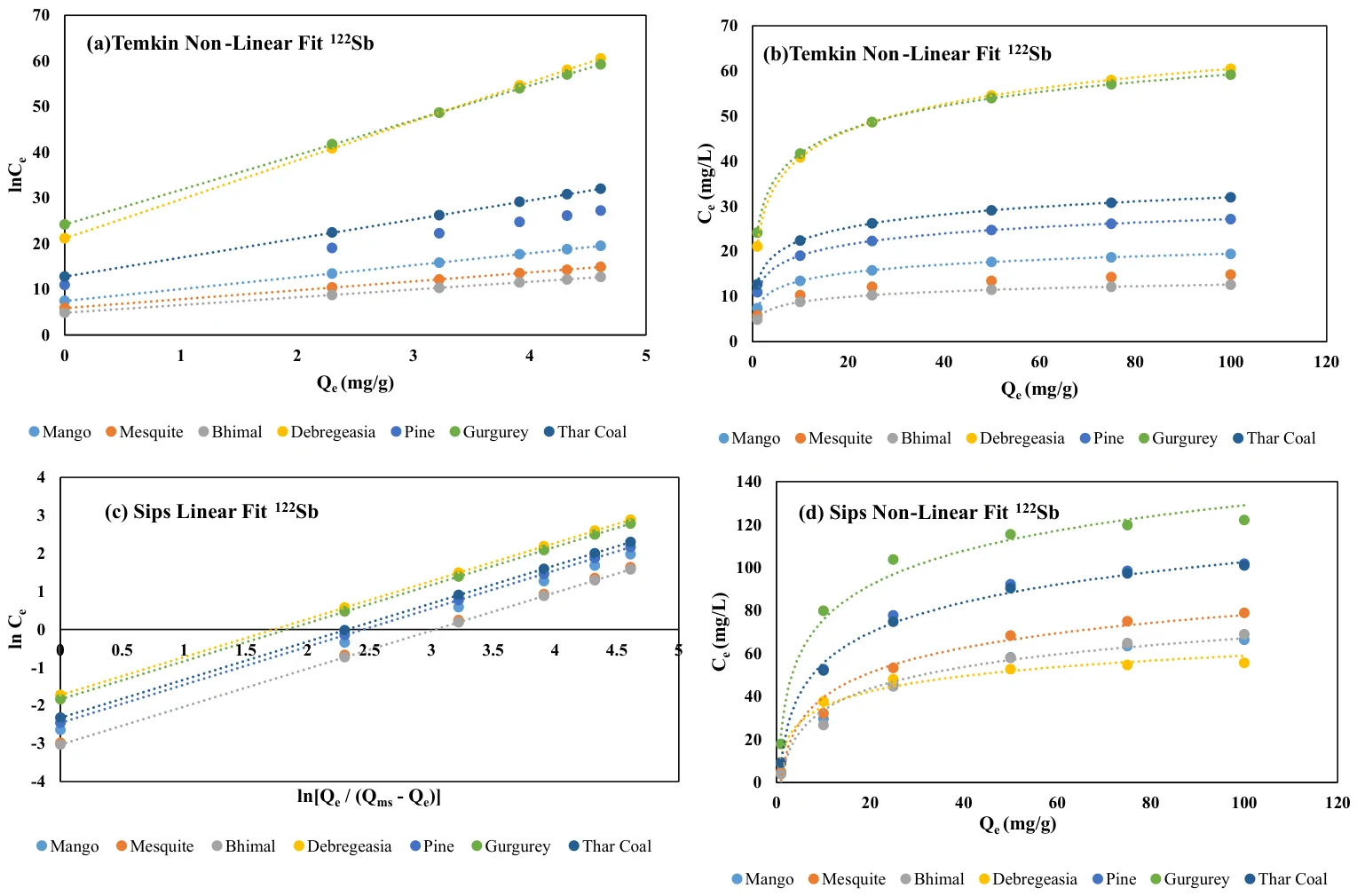
Linear and nonlinear plots for adsorption isotherms of ^122^Sb on activated carbons: (**a**,**b**) Temkin isotherm; (**c**,**d**) Sips isotherm.

The Sips model demonstrates more consistency with R^2^ = 0.99. β (heterogeneity factor) is close to 1 for all adsorbents, which implies that adsorption is in a strongly homogenous Langmuir monolayer. It indicates that active sites have high energy and form a single layer of ^122^Sb [99]. The most efficient adsorbents are Gurgurey and Pine, exhibiting adsorption capacities of 129 mg/g and 113.6 mg/g. The parameters for the Sips model are mentioned in Table S13. The graphs of Temkin and Sips for ^122^Sb are represented in Figure 16. 

### 3.5. Adsorption Capacities of Activated Carbons for ^137^Cs, ^134^Cs, ^124^Sb, and ^122^Sb

**Figure 17:**
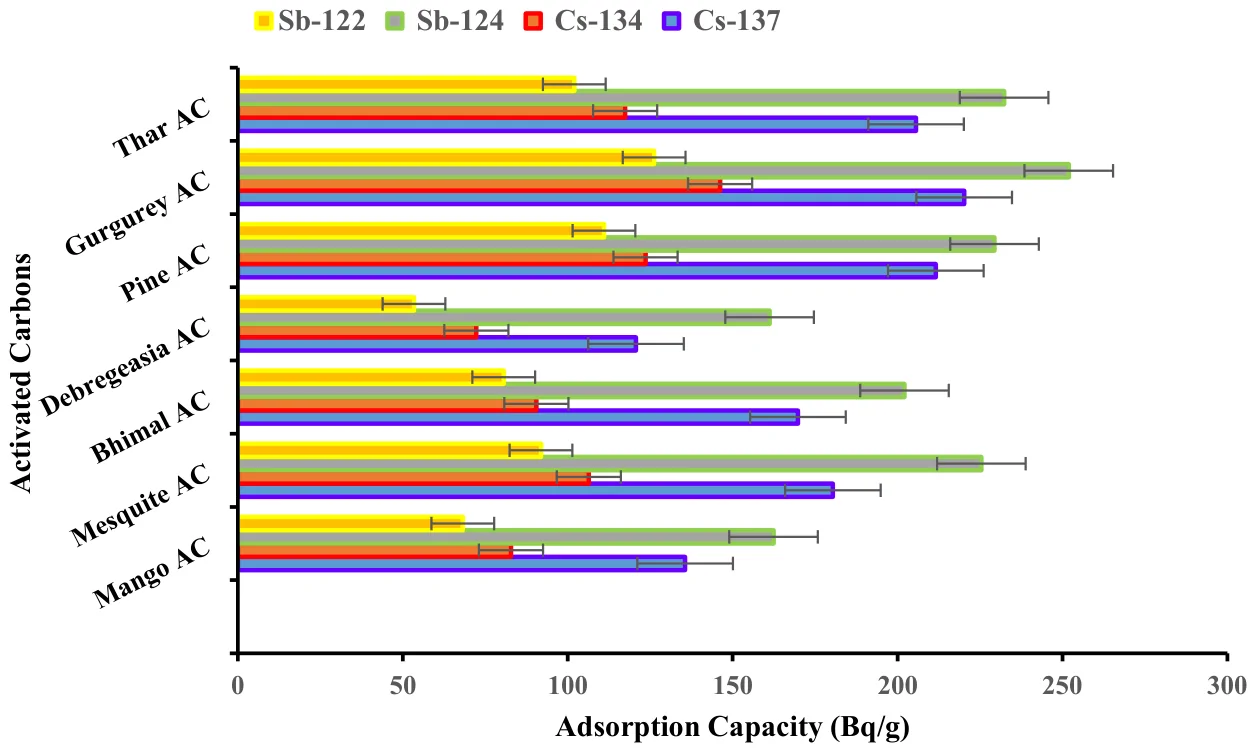
Comparison of activated carbons on the basis of their adsorption capacities for radionuclides (AC indicates activated carbon).

The results confirmed that the amount of adsorption is in the order ^124^Sb > ^137^Cs > ^134^Cs > ^122^Sb. Some of the factors affecting the adsorption capacity of the various adsorbents include pH, the presence of other cations that can adsorb, ionic strength, temperature, characteristics of the adsorbent materials, and the initial concentration of the adsorbate [100]. Thus, surface area and pore volume were essential for adsorption capacity, as found in the kinetics and equilibrium adsorption studies [101]. The trend of adsorption of the radionuclides in the present study can be attributed to the higher concentration of ^124^Sb ions than other radionuclides in the adsorbate [102]. The radionuclides with higher concentrations (92.3, 30.2, 95.8, and 5.8 Bq/mL of ^137^Cs, ^134^Cs, ^124^Sb and ^122^Sb) in radioactive wastewater compete more for the active sites of the activated carbon to get adsorbed. These results are in agreement with the factors highlighted by other authors [100,101] that the surface area, pore volume, and concentration of adsorbate affect the efficiency of adsorption. Each of the adsorbents’ adsorption capacities for ^137^Cs, ^134^Cs, ^124^Sb, and ^122^Sb are presented in Figure 17.

According to the results, the adsorption capacities of activated carbons for ^137^Cs obtained from woods of Mango, Mesquite, Bhimal, Debregeasia, Pine, Gurgurey, and Thar coal were 135.6, 180.4, 169.8, 120.7, 211.6, 220.2, and 205.6 Bq/gm, respectively. The greatest adsorption of ^137^Cs was achieved by Gurgurey AC (220.2 Bq/gm), while the minimum was observed by Debregeasia AC (120.7 Bq/gm), probably because it was not as porous as the other adsorbents used in this study. Like what was observed in the cases of ^137^Cs, the activated carbon derived from Gurgurey wood had a maximum adsorption capacity of 146 Bq/g for ^134^Cs. Thus, the carbon obtained with the use of Debregeasia wood had the lowest uptake capacity at 72. 3 Bq/gm, while the overall trend of adsorption was not as high as in the case of ^137^Cs. As illustrated in Figure 12, the activated carbon derived from Gurgurey obtained the highest adsorption of ^124^Sb at 251.9 Bq/gm, and the lowest was recorded in Debregeasia-based AC at 161.2 Bq/gm. For ^122^Sb, the ACs prepared from Mango, Mesquite, Bhimal, Debregeasia, Pine, Gurgurey, and Thar coal had maximum adsorption capacities of 68.2, 91.9, 80.6, 53 4, 111.0, 126.2, and 102 Bq/gm, respectively. Concluding, the activated carbon obtained from Gurgurey had the highest adsorption capacity for ^122^Sb (126.2 Bq/gm), while Debregeasia AC had the lowest capacity of 53.4 Bq/gm for ^122^Sb. These findings confirm that activated carbons derived from wood exhibited a high uptake of the radionuclides, with Gurgurey recording the highest adsorption capacities for all the tested isotopes.

For all four radionuclides (^137^Cs, ^134^Cs, ^124^Sb, and ^122^Sb), the adsorption capacities of the different activated carbons showed a similar pattern: high values at the start and finish of the sequence, with a noticeable dip in the middle. The activated carbon made from Debregeasia wood, which continuously demonstrated the lowest adsorption capacities among all tested radionuclides, is responsible for this dip. The physicochemical characteristics of the sorbents are the root cause of this pattern. Activated carbon derived from Debregeasia had the lowest fixed carbon content (33.9%) and the highest moisture (12.1%), volatile matter (47.3%), and ash (18.8%) contents. Reduced adsorption performance is the result of these factors, which also lead to a less porous structure and fewer active adsorption sites. Conversely, activated carbons made from Pine, Gurgurey, and Thar coal showed better proximate qualities, especially higher fixed carbon and lower moisture and ash contents, which are directly linked to higher sorption efficiencies. Therefore, the observed dip is not accidental; rather, it shows a direct relationship between the properties of the materials and how well they remove radionuclides.

### 3.6. Adsorption Mechanism

**Figure 18:**
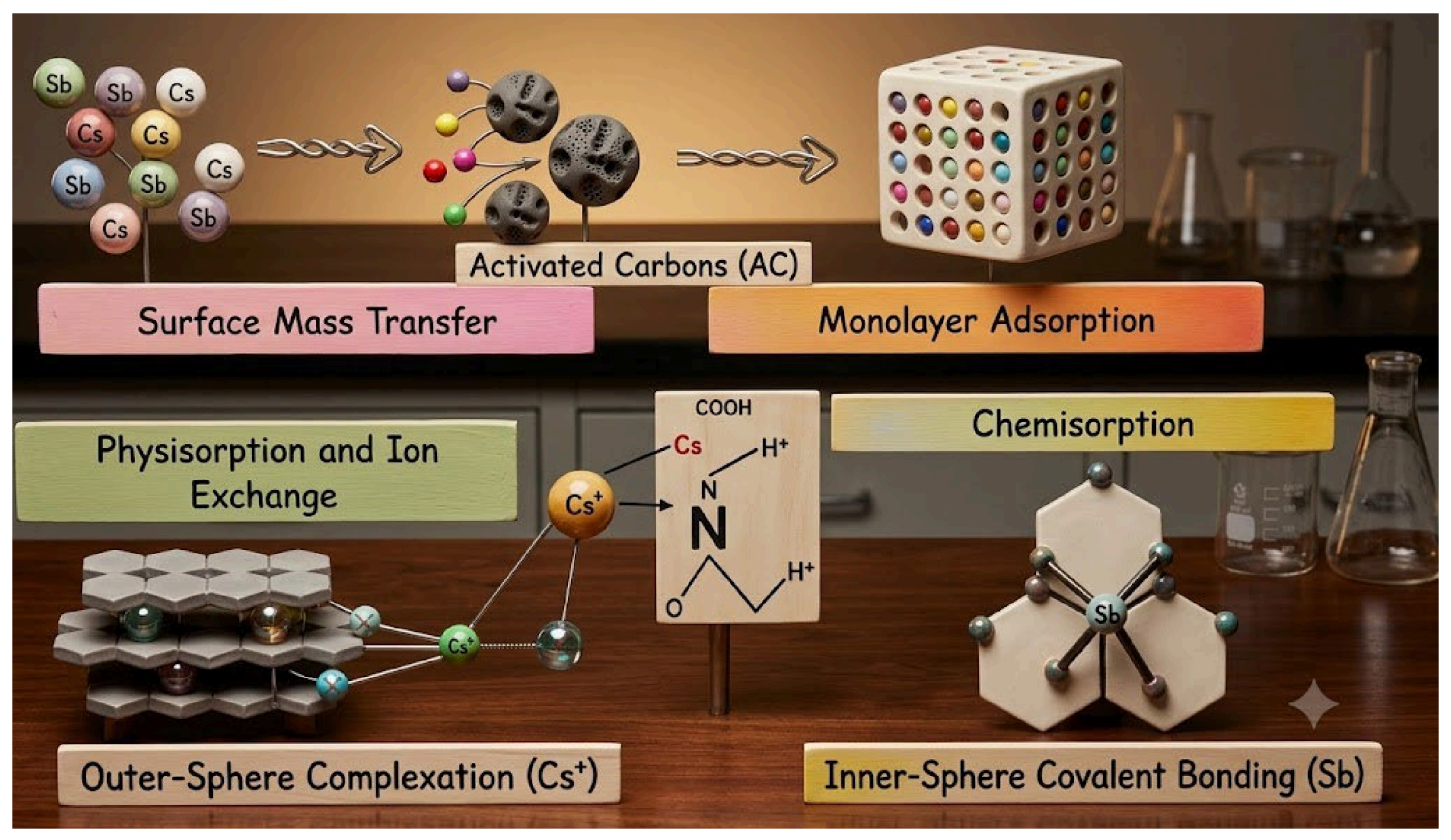
Schematic illustration of the underlying mechanisms for multi-nuclide adsorption onto engineered activated carbon. The adsorption pathway involves bulk-to-surface mass transfer followed by uniform monolayer coverage across active surface sites. Cesium (Cs 137/Cs 134) capture is driven by physio-sorption, outer-sphere complexation, and ion exchange interacting with oxygen-containing functional groups (carboxyl, phenolic, hydroxyl). Conversely, antimony (Sb 124, Sb 122) capture is governed by stable chemisorption through inner-sphere covalent bonding directly onto the carbon matrix. Figure 18 is an author-designed schematic illustration. Artificial-intelligence-assisted image generation/editing tools were used only for graphical visualization and layout improvement. All scientific content, experimental design, interpretation, and final verification were performed by the authors.

To explicitly map out the interfacial dynamics governing multi-nuclide remediation, a detailed schematic of the underlying mechanisms is provided in Figure 18. The adsorption process initiates via rapid bulk-to-surface mass transfer, where solvated radioisotopes migrate from the concentrated aqueous phase toward the boundary layer of the porous carbon matrices [103]. Supported by the high-density micro/mesoporous frameworks of Gurgurey wood and Thar coal, this fluid-to-solid mass transfer transitions into a uniform, monolayer distribution across active surface sites—a pathway strictly validated by the excellent fit of the Langmuir isotherm and pseudo-second-order kinetic models.

As visually elucidated in Figure 18, the chemical sequestration of the target radionuclides diverges into two distinct pathways depending on the ionic species. For the cesium isotopes (Cs 137 and Cs 134), uptake is predominantly driven by physio-sorption, outer-sphere complexation, and classic ion exchange [104]. The active oxygen-containing functional groups, including carboxyl (-COOH), phenolic, and hydroxyl species anchored on the carbonaceous skeleton, serve as the primary electrostatic nests for capturing the highly mobile single-charged ${\mathrm{Cs}}^{+}$ cations. On the contrary, the removal of antimony radioisotopes (Sb 124 and Sb 122) is mediated by an energetic chemisorption mechanism [105]. Rather than simple electrostatic attraction, antimony forms highly stable, immobile inner-sphere complexes through direct covalent bonding with the activated matrix [106]. On the contrary, the adsorption of ^124^Sb and ^122^Sb across activated carbons occurs in stable inner-sphere complexes [107]. The experimental values of q_e_ in PSO and Langmuir isotherms show the formation of a stable monolayer during the adsorption process [108]. Moreover, the porous surface of ACs (like Gurgurey and Thar coal) gives high-density functional groups on the surface, easing mass transfer and the retention of radionuclides at low equilibrium concentrations [109]. The detailed illustrative mechanism is highlighted in Figure 18. This mechanistic synergy provides a robust explanation for the high experimental adsorption capacities ($q_{e}$) maintained by these engineered materials even at low equilibrium concentrations, establishing them as effective barriers for nuclear wastewater treatment.

### 3.7. Comparison with Other Studies

To assert the efficacy of wood-based activated carbons, a comparison was conducted between previously existing adsorbents in the literature. Table 1 shows that the adsorption capacities of Gurgurey and Thar coal ACs show consistent and superior activity against other bio-based and natural adsorbents. This elite efficiency is attributed to the high porosity, density, and presence of functional groups on the hierarchical matrix. The physiochemical properties of the adsorbent ensured rapid mass transfer and complexation across active sites. The compatibility with Langmuir and PSO shows that the adsorbent is compatible and reliable for the treatment of radioactive waste.

## 4. Conclusions

In order to remove radionuclides (^137^Cs, ^134^Cs, ^124^Sb, and ^122^Sb) from low-level radioactive wastewater, this study assessed the adsorption potential of activated carbons modified with phosphoric acid and derived from various lignocellulosic biomass sources and Thar coal. The highest adsorption capacity was demonstrated by activated carbon made from Gurgurey wood, followed by Pine wood and Thar coal among the tested adsorbents. These materials’ superior performance was linked to their favorable proximate characteristics, especially their high fixed carbon percentage and low moisture, volatile, and ash content, all of which are crucial factors affecting adsorption behavior. The majority of activated carbons have phosphorus-containing functional groups, which improve chemical interaction with radionuclide species, according to FTIR analyses. Langmuir isotherms best fit the equilibrium data, indicating monolayer adsorption on homogeneous surfaces, while adsorption kinetics followed pseudo-second-order models, suggesting chemisorption. These results imply that well-designed coal-based and bio-based activated carbons provide viable and long-term solutions for nuclear wastewater treatment applications. To evaluate the long-term effectiveness and economic viability of these adsorbents, regeneration and reuse studies are crucial for future research. Field-level or pilot-scale testing in actual operational settings would offer useful confirmation and scalability information. Lastly, this approach will be more applicable for more comprehensive nuclear decontamination strategies if the adsorbent evaluation is expanded to include other common radionuclides like ^60^Co, ^90^Sr, and ^110m^Ag. With major ramifications for public health and environmental preservation, the current work, taken as a whole, provides a solid basis for the creation of economical, environmentally friendly materials for the safe handling of radioactive wastewater.

## Figures and Tables

**Table 1:** Comparison between ACs (this study) and other adsorbents in the literature.

Adsorbent	Radionuclide	q_max_ (mg/g)	Model (Isotherm/Kinetic)	Mechanism	Reference
Fabricated ACs (this study)	^137^Cs, ^134^Cs, ^122^Sb, ^124^Sb	232.3	Langmuir/PSO	Physisorption and chemisorption	NA
Bio-based activated carbon	^137^Cs	55.25	Langmuir/PSO	Ion-exchange/surface complexation	[110]
Graphene oxide (GO)	^60^Co, ^137^Cs	93.70	Langmuir/PSO	Strong chemisorption	[111]
Bentonite–alginate	^137^Cs	233.9–266.6	Langmuir/Fast-uptake	Ion exchange and interlayer trapping	[112]
Natural diatomite	^137^Cs, Ba^+2^	21.4	Freundlich/PSO	Electrostatic/ion exchange	[113]
Phosphate adsorbent	^90^Sr, ^137^Cs	12.5–45.0	Langmuir/PSO	Electrostatic interaction	[114]
Amidoxime-functionalized AC	U(VI)	66.35	Langmuir/PSO	Surface complexation	[115]
PEI-modified AC/Fe	U(VI)	15.3	Langmuir	Electrostatic/chelation	[116]

## Data Availability

The original contributions presented in this study are included in the article/Supplementary Material. Further inquiries can be directed to the corresponding author.

## Supplementary Materials

The following supporting information can be downloaded at: https://www.mdpi.com/article/10.3390/nano16161040/s1, Table S1: Proximate analysis determination of charcoals/coal and activated carbons; Table S2: Linear and nonlinear kinetic metrics for PFO and PSO for ^137^Cs; Table S3: PFO and PSO linear and non-linear kinetics for ^134^Cs; Table S4: PFO and PSO linear and non-linear kinetics for ^124^Sb; Table S5: PFO and PSO linear and non-linear kinetics for ^122^Sb; Table S6: Linear and non-linear Langmuir and Freundlich isotherms for ^137^Cs; Table S7: Linear and non-linear Langmuir and Freundlich isotherms for ^134^Cs; Table S8: Parameters calculated with Langmuir and Freundlich isotherm models for adsorption of ^124^Sb; Table S9: Parameters calculated with Langmuir and Freundlich isotherm models for adsorption of ^122^Sb; Table S10: Parameters calculated with Temkin and Sips isotherm models for adsorption of ^137^Cs; Table S11: Parameters calculated with Temkin and Sips isotherm models for adsorption of ^134^Cs; Table S12: Parameters calculated with Temkin and Sips isotherm models for adsorption of ^124^Sb. Table S13: Parameters calculated with Temkin and Sips isotherm models for adsorption of ^122^Sb.
