# Supplementary material for: Engineered Activated Carbons for Multi-Radionuclide Removal from Real Low-Level Radioactive Wastewater
Source: Nanomaterials (Basel). 2026 Aug 21;16(16):1040. doi: 10.3390/nano16161040 (PMC13516663; doi:10.3390/nano16161040)
Supplement: Supplementary file 1 [file nanomaterials-16-01040-s001.zip › nanomaterials-4374208-supplementary.pdf]

Supporting Information

Methodology

S1. Proximate Analysis

S1.1. Determination of Moisture Content

Moisture content (MC) was calculated in accordance with ASTM D2867-17. A total of 1 gram of activated carbon or precursor was accurately weighed in a Petri dish and then heated for 3 hours in a hot air oven at a temperature of 105°C. The samples were then quickly covered and left in the desiccator to cool to room temperature before weighing again. Calculations were made using the formula provided below (S1):

$$Moisture\ content\ (\%) = \frac{(C - D)}{(C - B)} \times 100 \tag{S1}$$

where

B = weight of Petri dish + lid;

C = weight of Petri dish + lid + sample (prior to drying of the sample);

D = weight of Petri dish + lid + sample (after drying of the sample).

S1.2. Determination of Volatile Matter

Volatile matter (VM) was determined by following ASTM D5832-98(2021). In total, 1 gm of pre-dried sample, as already performed following ASTM D2867-17, was added into a pre-weighed crucible along with a lid. The crucible containing the dried sample was closed with its lid and placed in a muffle furnace at 750 ˚C for 30 minutes. The dried sample was left to cool down at room temperature and weighed. Calculations were made using the formula provided below (S2):

$$Volatile\ Matter\ (\%) = \frac{(C - D)}{(C - B)} \times 100 \tag{S2}$$

where,

B= weight of crucible + cover;

C= weight of crucible + cover + sample;

D= weight of crucible + cover + devolatilized sample.

S1.3. Determination of Ash Content

Ash content (AC) was quantified using ASTM D2866- 11. A weight of about 1 g of the dried sample was put in a pre-weighted crucible and then ashed in a muffle furnace at 650 °C for 3 hours. When the sample temperature came down to room temperature, the sample was weighed. The formula is provided below (S3):

$$Ash\ Content\ (\%) = \frac{(D - B)}{(C - B)} \times 100 \tag{S3}$$

where

B= weight of crucible without lid;

C= weight of crucible + sample (before heating);

D= weight of crucible + sample (after heating).

S1.4. Determination of Fixed Carbon Content

The amount of fixed carbon (FC) was determined by calculating mass differences. The following formula can be used to find fixed carbon (S4):

$$Fixed\ Carbon\ (\%) = 100 - (\%Ash + \%Volatile\ matter) \tag{S4}$$

S2. Adsorption Kinetics

S2.1. Pseudo-First-Order Kinetics

At the end of the 19th century, the pseudo-first-order kinetics (K_1) derived empirical rate equation was introduced for the adsorption of malonic and oxalic acid onto charcoal. This is the earliest known equation for determining the rate of sorption in liquid-phase-based adsorption processes. The linearized equation for pseudo-first-order is mentioned below (S5):

$$ln(q_e - q_t) = ln(q_e) - k_1 \cdot t \tag{S5}$$

Plotting is between ln (q_e-q_t) vs time. The rate constant of pseudo-first-order is obtained from the slope of the plot constructed between ln ($q_e - q_t$) vs time (t).

The nonlinear form of pseudo-first-order is expressed as (S6):

$$q_t = q_e(1 - e^{-k_1 t}) \quad \text{(S6)}$$

S2.2. Pseudo-Second-Order Kinetics

Pseudo-second-order kinetics (K_2) was presented in the mid-80s. It became very popular after the study, in which a number of experimental results from the literature were compared and concluded that pseudo-second-order fits best to the experimental data . The majority of studies follow pseudo-second-order kinetics, which means K_2 has superiority over K_1. The linearized equation for pseudo-second-order is mentioned below (S7):

$$dq_t/dt = k_2 (q_e - q_t)^2 \quad \text{(S7)}$$

Plotting is between t/q_t vs t. The parameters q_e and K_2 can be determined from the slope and intercept of the plot.

Nonlinear pseudo-second-order kinetics is expressed as (S8):

$$q_t = \frac{k_2 q_e^2 t}{1 + k_2 q_e t} \quad \text{(S8)}$$

S3. Adsorption Isotherms

S3.1. Langmuir Isotherm Model

This model is based on the assumption that there is monolayer adsorption at definite localized sites with no interaction or hindrance between adjacent molecules. The type of adsorption is homogeneous, with each site having an equal affinity for the molecules to get attached to it. Moreover, there is no transfer of adsorbate on the surface of the adsorbent. When the active sites are filled, no further adsorption will occur, and a saturation point is achieved.

The linear form of the Langmuir isotherm can be expressed as (S9):

$$\frac{C_e}{q_e} = \left(\frac{1}{q}{}_m\right) \cdot C_e + \left(\frac{1}{q}{}_m KL\right) \quad \text{(S9)}$$

Plotting is between C_e/q_e vs C_e. However, the values of q_{max} and K_L were calculated from the intercept and slope of the graph.

The nonlinear form of the Langmuir isotherm can be expressed as (S10):

$$q_e = \frac{q_{max} K_L C_e}{1+K_L C_e} \qquad \text{(S10)}$$

S3.2. Freundlich Isotherm Model

The Freundlich model is applied to adsorption processes based on adsorbents with heterogeneous surfaces and multilayer adsorption. There is an interaction between the attached molecules on the adsorbent. This isotherm is an empirical equation, and its linear form is mentioned below (S11):

$$\log(q_e) = \log(K_F) + \left(\frac{1}{n}\right) \cdot \log(C_e) \qquad \text{(S11)}$$

However, "1/n" also tells about the relative distribution of the energy and the heterogeneity of the adsorbate sites. Although "n" indicates linear adsorption if n is equal to 1 (n=1), if it is less than 1 (n < 1), it is chemisorption, while adsorption is physical if it is greater than 1 (n > 1). The plot is plotted between log q_e vs log C_e, where 1/n is the slope, and ln K_F is the intercept.

The nonlinear Freundlich isotherm is expressed as (S12):

$$q_e = K_f C^{1/n} \qquad \text{(S12)}$$

S3.3. Temkin Isotherm Model

The Temkin model demonstrates the heat of adsorption by a linear function rather than a log function for surface coverage. The linear form of the Temkin isotherm is expressed as (S13):

$$Q_e = B \ln A_T + B \ln C_e \qquad \text{(S13)}$$

The nonlinear form is expressed as (S14):

$$Q_e = B \ln(A_T .\ C_e) \qquad \text{(S14)}$$

where is the amount of adsorbed solute per unit mass of adsorbent (mg/g). $\mathbf{C_e}$ is the equilibrium concentration of the adsorbate (mg/L). $\mathbf{A_T}$ is the Temkin isotherm equilibrium binding constant (L/mg), and B is a constant related to the heat of adsorption (J/mol).

S3.4. Sips Isotherm Model

The Sips model is a mix of Langmuir and Frendluich isotherms and is applied to a mix of adsorptive materials. It rules out the limitation of high-concentration solutions. The linear form of the Sips isotherm model is expressed as (S15):

$$Ln \left(\frac{Qe}{Qms-Qe}\right) = \beta_s \ln C_e + \ln a_s \qquad \text{(S15)}$$

The nonlinear form of the Sips isotherm is expressed as (S16):

$$Q_e = \frac{Qms\,.as.Cs\ \beta s}{1+as.as.Cs\ \beta s} \qquad \text{(S16)}$$

where Q_{ms} is the Sips maximum adsorption capacity in mg/g. a_s is the Sips isotherm constant related to the energy of adsorption (L/mg). β_s is the Sips heterogeneity factor. When $\beta_s = 1$, the equation reduces to the Langmuir isotherm.

S4. Results and Discussion

Table S1. Proximate analysis determination of charcoals/coal and activated carbons.

	**Charcoal/Coal**				**Activated Carbon**			
	**Moisture Content**	**Volatile Matter**	**Ash Content**	**Fixed Carbon**	**Moisture Content**	**Volatile Matter**	**Ash Content**	**Fixed Carbon**
**Mango wood**	6.5	25.8	3.8	70.4	5.3	21.2	6.8	72
**Mesquite wood**	3.4	42.3	4.6	58.5	2.4	35.6	8.3	56.1
**Bhimal wood**	7.8	48.5	5.4	53.1	6.2	40.2	10.1	49.7
**Debregeasia wood**	15.3	53.6	13.5	46.1	12.1	47.3	18.8	33.9
**Pine wood**	4.8	25.2	3.3	71.5	3.8	15.3	5.2	79.5
**Gurgurey wood**	5.4	39.4	2.1	32.9	4.2	12	3.7	84.3
**Thar coal**	43.3	56.3	4.09	39.61	4.8	10.7	11.47	77.8

Table S2. Linear and nonlinear kinetic metrics for PFO and PSO for ^{137}Cs.

**Adsorbents**	**Q_e (exp)**	**PFO**								**PSO**								
		**Linear Kinetics**			**Nonlinear Kinetics**					**Linear Kinetics**				**Nonlinear Kinetics**				
		**R^2**	**K_1 (min^{-1})**	**q_e (Calc)**	**q_e (Calc)**	**K_1 (min^{-1})**	**R^2**	**ARE %**	**SSE**	**R^2**	**K_2 (min^{-1})**	**Q_e (Calc)**	**h**	**q_e (Calc)**	**K_2**	**R^2**	**ARE %**	**SSE**
**Mango**	135.6	0.23	0.0055	26.10	12.85	0.021	0.23	2.28	4.82	0.966	0.000060	140.8	1.19	45.52	0.00246	0.998	**0.95**	**1.35**
**Mesquite**	180.4	0.59	0.0084	5.58	25.4	0.015	0.59	1.27	3.15	0.989	0.000010	185.2	0.35	50.12	0.00339	0.999	0.61	**0.88**

**Bhimal**	169.8	0.57	0.0084	14.13	18.22	0.011	0.57	3.42	1.35	0.941	0.000012	172.4	0.35	33.42	0.00155	0.985	**0.78**	**1.22**
**Debregeasia**	120.7	0.97	0.0143	14.39	48.6	0.028	0.97	1.58	1.49	0.988	0.000052	123.5	0.79	104.39	0.00006	0.998	0.58	**0.42**
**Pine**	211.6	0.88	0.0084	5.58	38.45	0.019	0.88	2.11	1.37	0.984	0.000008	217.4	0.35	54.01	0.00183	0.997	**0.42**	**0.35**
**Gurgurey**	220.2	0.76	0.0069	17.58	30.15	0.014	0.76	1.95	0.81	0.993	0.000027	227.3	1.37	50.58	0.00084	0.978	**0.36**	**0.28**
**Thar coal**	205.6	0.93	-0.0079	22.91	40.22	0.012	0.93	2.44	1.60	0.989	0.000034	208.3	1.46	49.54	0.00061	0.975	**0.63**	**0.56**

Table S3. PFO and PSO linear and nonlinear kinetics for ^{134}Cs.

**Adsorbents**	**Q_e (exp)**	**PFO**								**PSO**							
		**Linear Kinetics**			**Nonlinear Kinetics**					**Linear Kinetics**				**Nonlinear Kinetics**			
		**R^2**	**K_1 (min^{-1})**	**q_e (Calc)**	**q_e (Calc)**	**K_1 (min^{-1})**	**R^2**	**ARE %**	**SSE**	**R^2**	**K_2 (min^{-1})**	**Q_e (Calc)**	**h**	**q_e (Calc)**	**K_2**	**R^2**	**ARE %**	**SSE**
**Mango**	82.8	0.03	0.0127	1.13	80.2	0.0154	0.894	2.28	4.12	0.966	0.000060	140.8	1.19	83.9	0.00031	0.996	0.72	0.84
**Mesquite**	106.4	0.16	0.0196	1.76	102.8	0.0215	0.912	1.27	3.88	0.989	0.000010	185.2	0.35	107.5	0.00028	0.998	0.58	0.88
**Bhimal**	90.5	0.77	0.0221	1.86	88.4	0.0238	0.948	3.42	2.45	0.941	0.000012	172.4	0.35	92.1	0.00035	0.994	0.81	0.65
**Debregeasia**	72.3	0.01	0.0182	1.51	69.5	0.0192	0.97	1.58	5.02	0.988	0.000052	123.5	0.79	73.8	0.00042	0.997	0.63	0.58
**Pine**	123.6	0.5	0.0221	1.86	119.3	0.0241	0.885	2.11	3.16	0.984	0.000008	217.4	0.35	124.7	0.00022	0.999	0.39	0.42
**Gurgurey**	146.2	0.84	0.0205	1.53	143.1	0.0224	0.952	1.95	2.18	0.993	0.000027	227.3	1.37	147.8	0.00025	0.999	0.42	0.31
**Thar coal**	117.4	0.04	0.0205	1.44	114.2	0.0218	0.876	2.44	5.84	0.989	0.000034	208.3	1.46	119.5	0.00029	0.995	0.87	0.98

Table S4. PFO and PSO linear and nonlinear kinetics for [124]Sb.

Adsorbents	Q_e (exp)	PFO								PSO								
		Linear Kinetics			Nonlinear Kinetics					Linear Kinetics				Nonlinear Kinetics				
		R^2	K_1 (min^{-1})	q_e (Calc)	q_e (Calc)	K_1 (min^{-1})	R^2	ARE %	SSE	R^2	K_2 (min^{-1})	Q_e (Calc)	h	q_e (Calc)	K_2	R^2	ARE %	SSE
**Mango**	162.4	0.28	0.0046	6.23	158.4	0.0128	0.908	2.28	4.82	0.95	0.00008	166.7	2.09	164.8	0.00018	0.992	0.84	1.12
**Mesquite**	225.4	0.27	0.0138	54.19	220.1	0.0184	0.915	1.85	5.54	0.99	0.0001	227.3	4.93	228.4	0.00015	0.997	0.42	0.78
**Bhimal**	202.1	0.64	0.0092	44.67	198.5	0.0152	0.932	1.45	3.92	0.98	0.00012	204.1	4.93	163.7	0.00021	0.995	0.67	1.05
**Debregeasia**	161.2	0.29	0.0118	5.16	156.2	0.0165	0.895	2.52	6.18	0.98	0.00008	163.9	2.20	205.3	0.00024	0.994	0.71	0.85
**Pine**	229.4	0.80	0.0124	54.19	224.8	0.0195	0.941	1.68	4.22	0.99	0.00009	232.6	4.93	232.1	0.00014	0.998	0.38	0.56
**Gurgurey**	251.9	0.85	0.0023	9.71	248.3	0.0210	0.958	1.15	3.14	0.99	0.00003	256.4	1.73	254.9	0.00011	0.999	0.31	0.29
**Thar coal**	102	0.75	0.0023	8.18	98.4	0.0142	0.882	2.94	7.35	0.99	0.00016	105.3	1.73	104.2	0.00028	0.996	0.82	0.92

Table S5. PFO and PSO linear and nonlinear kinetics for [122]Sb.

Adsorbents	Q_e (exp)	PFO								PSO								
		Linear Kinetics			Nonlinear Kinetics					Linear Kinetics				Nonlinear Kinetics				
		R^2	K_1 (min^{-1})	q_e (Calc)	q_e (Calc)	K_1 (min^{-1})	R^2	ARE %	SSE	R^2	K_2 (min^{-1})	Q_e (Calc)	h	q_e (Calc)	K_2	R^2	ARE %	SSE
**Mango**	68.2	0.48	0.0046	6.83	6.83	0.0128	0.908	2.28	4.82	0.955	0.00012	72.5	0.63	72.5	0.00012	0.992	0.84	1.12
**Mesquite**	91.9	0.82	0.0027	3.33	3.33	0.0184	0.915	1.85	5.54	0.989	0.00006	95.2	0.57	95.2	0.00006	0.997	0.42	0.78
**Bhimal**	80.6	0.86	0.0027	2.96	2.96	0.0152	0.932	1.45	3.92	0.984	0.00009	82	0.57	82	0.00009	0.995	0.67	1.05

**Debregeasia**	53.4	0.98	0.0111	5.8	5.8	0.0165	0.895	2.52	6.18	1	0.00007	56.2	0.2	56.2	0.00007	0.994	0.71	0.85
**Pine**	111	0.82	0.0027	2.96	2.96	0.0195	0.941	1.68	4.22	0.989	0.00004	113.6	0.57	113.6	0.00004	0.998	0.38	0.56
**Gurgurey**	126.2	0.72	0.011	1.34	1.34	0.021	0.958	1.15	3.14	0.996	0.00001	128.2	0.22	128.2	0.00001	0.999	0.31	0.29
**Thar coal**	232.3	0.68	0.011	1.03	1.03	0.0142	0.882	2.94	7.35	0.922	0	232.6	0.22	232.6	0	0.996	0.82	0.92

Table S6. Linear and nonlinear Langmuir and Frendluich isotherms for ^{137}Cs.

		**Langmuir Isotherm**							**Freundlich Isotherm**						
**Adsorbents**	**Q_e (exp)**	**Linear**				**Nonlinear**			**Linear**				**Nonlinear**		
		**Q_{max}**	**b**	**R_L**	**R^2**	**Q_{max} (mg/g)**	**b (L/mg)**	**R^2**	**K_F**	**n**	**R^2**	**1/n**	**K_f**	**n**	**R^2**
Mango	135.6	138.9	0.0165	0.9838	0.99	137.24	0.0172	0.998	2.314	0.6112	0.98	1.636	2.451	0.628	0.988
Mesquite	180.4	181.8	0.0056	0.9945	0.99	182.15	0.0058	0.999	0.656	1.3400	0.99	0.746	0.692	1.352	0.992
Bhimal	169.8	172.4	0.0206	0.9798	0.99	171.02	0.0215	0.999	2.640	0.6236	0.99	1.603	2.784	0.641	0.995
Debregeasia	120.7	125.0	0.0137	0.9865	0.99	124.6	0.0141	0.997	2.047	0.6252	0.99	1.599	2.112	0.635	0.991
Pine	211.6	212.8	0.0387	0.9628	0.99	213.45	0.0395	0.998	3.519	0.6001	0.98	1.667	3.61	0.612	0.989
Gurgurey	220.2	222.2	0.0613	0.9422	0.99	221.88	0.0628	0.999	4.301	0.5297	0.96	1.888	4.425	0.538	0.975
Thar coal	205.6	208.3	0.0333	0.9678	0.99	207.52	0.0342	0.998	3.277	0.6197	0.98	1.614	3.398	0.624	0.986

Table S7. Linear and nonlinear Langmuir and Frendluich isotherms for ^{134}Cs.

		Langmuir Isotherm							Freundlich Isotherm						
Adsorbents	Q_e (exp)	Linear				Nonlinear			Linear				Nonlinear		
		Q_{max}	b	R_L	R^2	Q_{max} (mg/g)	b (L/mg)	R^2	K_F	n	R^2	1/n	K_f	n	R^2
Mango	82.8	84.0	0.0339	0.9672	0.99	84	0.0339	0.99	1.968A	0.7399	0.99	1.352	1.968	1.352	0.99
Mesquite	106.4	107.5	0.0709	0.9338	0.99	107.5	0.0709	0.99	3.124	0.5791	0.99	1.727	3.124	1.727	0.99
Bhimal	90.5	95.2	0.0338	0.9673	0.99	95.2	0.0338	0.99	1.962	0.7800	0.99	1.282	1.962	1.282	0.99
Debregeasia	72.3	77.5	0.0669	0.9373	0.99	77.5	0.0669	0.99	2.921	0.4693	0.98	2.131	2.921	2.131	0.98
Pine	123.6	125.0	0.0507	0.9518	0.99	125	0.0507	0.99	2.524	0.8041	0.99	1.244	2.524	1.244	0.99
Gurgurey	146.2	149.3	0.1001	0.9090	0.99	149.3	0.1001	0.99	3.553	0.7312	0.99	1.368	3.553	1.368	0.99
Thar coal	117.4	119.0	0.0517	0.9509	0.00	119	0.0517	0.99*	2.595	0.7474	0.99	1.338	2.595	1.338	0.99

Table S8. Parameters calculated with Langmuir and Freundlich isotherm models for adsorption of ^{124}Sb.

		Langmuir Isotherm							Freundlich Isotherm						
Adsorbents	Q_e (exp)	Linear				Nonlinear			Linear				Nonlinear		
		Q_{max}	b	R_L	R^2	Q_{max} (mg/g)	b (L/mg)	R^2	K_F	n	R^2	1/n	K_f	n	R^2
Mango	162.4	166.7	0.0265	0.9742	0.99	166.7	0.0265	0.99	3.124	0.5453	0.95	1.352	3.124	1.834	0.95
Mesquite	225.4	227.3	0.0054	0.9947	0.99	227.3	0.0054	0.99	0.502	1.6963	0.99	1.727	0.502	0.59	0.99
Bhimal	202.1	204.1	0.0153	0.985	0.999	204.1	0.0153	0.999	1.923	0.8581	0.99	1.282	1.923	1.165	0.99

Debregeasia	161.2	166.7	0.0679	0.9364	0.99	166.7	0.0679	0.99	4.689	0.3647	0.90	2.131	4.689	2.742	0.9
Pine	229.4	232.6	0.034	0.9671	0.99	232.6	0.034	0.99	3.196	0.6907	0.99	1.244	3.196	1.448	0.99
Gurgurey	251.9	256.4	0.0521	0.9505	0.99	256.4	0.0521	0.99	3.7	0.7153	0.99	1.368	3.7	1.398	0.99
Thar coal	232.3	238.1	0.0259	0.9747	0.99	238.1	0.0259	0.99	2.592	0.8165	0.99	1.338	2.592	1.225	0.99

Table S9. Parameters calculated with Langmuir and Freundlich isotherm models for adsorption of ^{122}Sb.

		**Langmuir Isotherm**							**Freundlich Isotherm**						
**Adsorbents**	**Q_e (exp)**	**Linear**				**Nonlinear**			**Linear**				**Nonlinear**		
		**Q_{max}**	**b**	**R_L**	**R^2**	**Q_{max} (mg/g)**	**b (L/mg)**	**R^2**	**K_F**	**n**	**R^2**	**1/n**	**K_f**	**n**	**R^2**
Mango	68.2	71.4	0.0713	0.9335	0.99	71.4	0.0713	0.99	2.720	0.5237	0.99	1.910	2.72	1.91	0.99
Mesquite	91.9	95.2	0.0510	0.9515	0.99	95.2	0.051	0.99	2.219	0.8082	0.99	1.237	2.219	1.237	0.99
Bhimal	80.6	82.0	0.0483	0.9540	0.99	82	0.0483	0.99	2.152	0.7517	0.99	1.330	2.152	1.33	0.99
Debregeasia	53.4	58.8	0.1780	0.8489	0.99	58.8	0.178	0.99	3.619	0.2809	0.96	3.560	3.619	3.56	0.96
Pine	111.0	113.6	0.0868	0.9201	0.99	113.6	0.0868	0.99	2.964	0.7640	0.99	1.309	2.964	1.309	0.99
Gurgurey	126.2	129.9	0.1599	0.8621	0.99	129.9	0.1599	0.99	3.856	0.7361	0.99	1.359	3.856	1.359	0.99
Thar coal	102.0	105.3	0.0981	0.9106	0.99	105.3	0.0981	0.99	3.204	0.6295	0.99	1.589	3.204	1.589	0.99

Table S10. Parameters calculated with Temkin and Sips isotherm models for adsorption of ^{137}Cs.

		Temkin Isotherm						Sips Isotherm							
		Linear			Nonlinear			Linear				Nonlinear			
**Adsorbents**	**Q_e (exp)**	**B**	**A_T (L/mg)**	**Q_{ms} (mg/g)**	**A_T (L/mg)**	**B**	**R^2**	**Q_{ms} (mg/g)**	**a_s(L/mg)**	**β_s**	**R^2**	**Q_{ms} (mg/g)**	**a_s(L/mg)**	**β_s**	**R^2**
**Mango**	135.6	27.84	0.792	140.2	0.842	28.45	0.985	137.4	0.017	0.98	0.999	137.4	0.017	0.98	0.999
**Mesquite**	180.4	43.15	0.198	183.5	0.215	45.12	0.991	182.2	0.006	1.02	0.999	182.2	0.006	1.02	0.999
**Bhimal**	169.8	33.52	0.854	174.1	0.912	34.66	0.99	171.5	0.021	0.97	0.999	171.5	0.021	0.97	0.999
**Debregeasia**	120.7	25.44	0.712	126.8	0.764	26.18	0.988	124.8	0.014	0.99	0.998	124.8	0.014	0.99	0.998
**Pine**	211.6	41.92	1.085	214.5	1.154	42.85	0.982	213.6	0.039	0.96	0.999	213.6	0.039	0.96	0.999
**Gurgurey**	220.2	47.11	1.402	224.8	1.482	48.33	0.974	221.9	0.063	0.94	0.999	221.9	0.063	0.94	0.999
**Thar coal**	205.6	40.08	0.985	210.5	1.055	41.22	0.984	207.8	0.034	0.98	0.999	207.8	0.034	0.98	0.999

Table S11. Parameters calculated with Temkin and Sips isotherm models for adsorption of ^{134}Cs.

		Temkin Isotherm						Sips Isotherm							
		Linear			Nonlinear			Linear				Nonlinear			
**Adsorbents**	**Q_e (exp)**	**B**	**A_T (L/mg)**	**Q_{ms} (mg/g)**	**A_T (L/mg)**	**B**	**R^2**	**Q_{ms} (mg/g)**	**a_s(L/mg)**	**β_s**	**R^2**	**Q_{ms} (mg/g)**	**a_s(L/mg)**	**β_s**	**R^2**
**Mango**	135.6	21.42	1.15	0.98	21.85	1.18	0.99	84.2	0.033	1.02	0.99	84	0.0339	1	0.99
**Mesquite**	180.4	19.55	3.42	0.98	19.82	3.51	0.99	107.8	0.07	0.98	0.99	107.5	0.0709	1	0.99
**Bhimal**	169.8	23.18	1.22	0.98	23.45	1.25	0.99	95.5	0.033	1.04	0.99	95.2	0.0338	1	0.99
**Debregeasia**	120.7	14.88	3.58	0.97	15.12	3.65	0.98	77.9	0.065	0.95	0.98	77.5	0.0669	1	0.99
**Pine**	211.6	28.65	1.85	0.98	28.92	1.91	0.99	125.4	0.051	0.99	0.99	125	0.0507	1	0.99
**Gurgurey**	220.2	27.15	4.85	0.98	27.42	4.92	0.99	149.8	0.099	0.97	0.99	149.3	0.1001	1	0.99
**Thar coal**	205.6	26.88	1.92	0.98	27.15	1.98	0.99	119.5	0.051	1.01	0.99	119	0.0517	1	0.99

Table S12. Parameters calculated with Temkin and Sips isotherm models for adsorption of ^{124}Sb.

		Temkin Isotherm						Sips Isotherm							
		Linear			Nonlinear			Linear				Nonlinear			
**Adsorbents**	**Q_e (exp)**	**B**	**A_T (L/mg)**	**Q_{ms} (mg/g)**	**A_T (L/mg)**	**B**	**R^2**	**Q_{ms} (mg/g)**	**a_s(L/mg)**	**β_s**	**R^2**	**Q_{ms} (mg/g)**	**a_s(L/mg)**	**β_s**	**R^2**
**Mango**	42.15	0.48	0.94	42.85	0.52	0.95	167.2	0.026	0.98	0.99	166.7	0.0265	1	0.99	42.15
**Mesquite**	68.32	0.12	0.98	69.15	0.15	0.99	228.1	0.005	1.05	0.99	227.3	0.0054	1	0.99	68.32
**Bhimal**	55.42	0.28	0.98	56.24	0.31	0.99	204.8	0.015	1.02	0.99	204.1	0.0153	1	0.99	55.42
**Debregeasia**	34.18	1.15	0.89	34.92	1.22	0.9	167.5	0.067	0.92	0.99	166.7	0.0679	1	0.99	34.18
**Pine**	60.12	0.65	0.98	61.05	0.72	0.99	233.5	0.033	0.99	0.99	232.6	0.034	1	0.99	60.12
**Gurgurey**	64.85	0.98	0.98	65.72	1.05	0.99	257.2	0.051	0.97	0.99	256.4	0.0521	1	0.99	64.85
**Thar coal**	61.54	0.45	0.98	62.38	0.49	0.99	238.9	0.025	1.03	0.99	238.1	0.0259	1	0.99	61.54

Table S13. Parameters calculated with Temkin and Sips isotherm models for adsorption of ^{122}Sb.

		**Temkin Isotherm**						**Sips Isotherm**							
		**Linear**			**Nonlinear**			**Linear**				**Nonlinear**			
**Adsorbents**	**Q_e (exp)**	**B**	**A_T (L/mg)**	**Q_{ms} (mg/g)**	**A_T (L/mg)**	**B**	**R^2**	**Q_{ms} (mg/g)**	**a_s(L/mg)**	**β_s**	**R^2**	**Q_{ms} (mg/g)**	**a_s(L/mg)**	**β_s**	**R^2**
**Mango**	68.2	16.42	2.54	0.98	16.85	2.62	0.99	71.8	0.071	1.02	0.99	71.4	0.0713	1	0.99
**Mesquite**	91.9	19.85	1.88	0.98	20.12	1.95	0.99	95.8	0.05	0.98	0.99	95.2	0.051	1	0.99
**Bhimal**	80.6	18.12	1.62	0.98	18.45	1.68	0.99	82.5	0.048	1.03	0.99	82	0.0483	1	0.99
**Debregeasia**	53.4	11.55	8.42	0.97	11.82	8.55	0.98	59.2	0.175	0.94	0.99	58.8	0.178	1	0.99
**Pine**	111	22.18	3.45	0.98	22.45	3.52	0.99	114.1	0.086	0.99	0.99	113.6	0.0868	1	0.99
**Gurgurey**	126.2	23.42	7.55	0.98	23.75	7.62	0.99	130.4	0.158	0.97	0.99	129.9	0.1599	1	0.99
**Thar coal**	102	20.88	4.12	0.98	21.15	4.18	0.99	105.8	0.097	1.01	0.99	105.3	0.0981	1	0.99
